# Current Awareness on Comparative and Functional Genomics

**DOI:** 10.1002/cfg.226

**Published:** 2003-02


**Comparative and Functional Genomics**
*Comp Funct Genom* 2003; 4: 161–168
Published online in Wiley InterScience (www.interscience.wiley.com). **DOI** 10.1002/cfg.226

### Current awareness on comparative and functional genomics

### 1 Reviews & symposia

2002. Special issue: Genetic Epidemiology and Microarrays. *Genet Epidemiol* **23:** (1)
2002. Special issue: NEIBank. *Mol Vis* **8:** (21)
Armstrong SA, Hsieh JJD, Korsmeyer SJ. 2002. Genomic approaches to the pathogenesis and treatment of acute lymphoblastic leukemias. *Curr Opin Hematol* **9:** (4) 339.
Beroza P, Villar HO, Wick MM, Martin GR. 2002. Chemoproteomics as a basis for post-genomic drug discovery (Review). *Drug Discov Today* **7:** (15) 807.
Betts JC. 2002. Transcriptomics and proteomics: Tools for the identification of novel drug targets and vaccine candidates for tuberculosis. *IUBMB Life* **53:** (4-5) 239.
Botcherby M. 2002. Harvesting the mouse genome (Review). *Comp Funct Genom* **3:** (4) 319.
Brizuela L, Richardson A, Marsischky G, Labaer J. 2002. The FLEXGene repository: Exploiting the fruits of the genome projects by creating a needed resource to face the challenges of the post-genomic era. *Arch Med Res* **33:** (4) 318.
Burbaum J, Tobal GM. 2002. Proteomics in drug discovery. *Curr Opin Chem Biol* **6:** (4) 427.
Grunenfelder B, Winzeler EA. 2002. Treasures and traps in genome-wide data sets: Case examples from yeast. *Nat Rev Genet* **3:** (9) 653.
Heller MJ. 2002. DNA microarray technology: Devices, systems, and applications. *Annu Rev Biomed Eng* **4:** 129.
Heller S. 2002. Application of physiological genomics to the study of hearing disorders (Review). *J Physiol (Lond)* **543:** (1) 3.
Hochstrasser DF, Sanchez JC, Appel RD. 2002. Proteomics and its trends facing nature's complexity: Review. *Proteomics* **2:** (7) 807.
Holtorf H, Guitton MC, Reski R. 2002. Plant functional genomics (Review). *Naturwissenschaften* **89:** (6) 235.
Jordan IK, Rogozin IB, Wolf YI, Koonin EV. 2002. Microevolutionary genomics of bacteria. *Theor Popul Biol* **61:** (4) 435.
Junter GA, Coquet L, Vilain S, Jouenne T. 2002. Immobilized-cell physiology: Current data and the potentialities of proteomics. *Enzyme Microb Technol* **31:** (3) 201.
Kennedy S. 2002. The role of proteomics in toxicology: Identification of biomarkers of toxicity by protein expression analysis (Review). *Biomarkers* **7:** (4) 269.
Kleerebezem M, Boels IC, Groot MN, Mierau I, Sybesma W, Hugenholtz J. 2002. Metabolic engineering of *Lactococcus lactis*: The impact of genomics and metabolic modelling. *J Biotechnol* **98:** (2-3) 199.
Lopez-Otin C, Overall CM. 2002. Protease degradomics: A new challenge for proteomics. *Nat Rev Mol Cell Biol* **3:** (7) 509.
Lorenz MC. 2002. Genomic approaches to fungal pathogenicity. *Curr Opin Microbiol* **5:** (4) 372.
MacGregor PF, Squire JA. 2002. Application of microarrays to the analysis of gene expression in cancer (Review). *Clin Chem* **48:** (8) 1170.
McCormack GP, Clewley JP. 2002. The application of molecular phylogenetics to the analysis of viral genome diversity and evolution (Review). *Rev Med Virol* **12:** (4) 221.
Panek H, O'Brian MR. 2002. A whole genome view of prokaryotic haem biosynthesis (Review). *Microbiology* **148:** (8) 2273.
Quinn-Senger KE, Ramachandran R, Rininger JA, Kelly KM, Lewin DA. 2002. Staking out novelty on the genomic frontier. *Curr Opin Chem Biol* **6:** (4) 418.
Schofield D, Triche TJ. 2002. cDNA microarray analysis of global gene expression in sarcomas. *Curr Opin Oncol* **14:** (4) 406.
Schultze JL, Bohlen H. 2002. Influence of genomics on cancer vaccine development: From guess to prediction. *Curr Pharm Design* **8:** (19) 1735.
Shimamoto K, Kyozuka J. 2002. Rice as a model for comparative genomics of plants. *Annu Rev Plant Biol* **53:** 399.
Srinivas PR, Verma M, Zhao YM, Srivastava S. 2002. Proteomics for cancer biomarker discovery (Review). *Clin Chem* **48:** (8) 1160.
Tefferi A, Bolander ME, Ansell SM, Wieben ED, Spelsberg TC. 2002. Primer on medical genomics. Part III: Microarray experiments and data analysis. *Mayo Clin Proc* **77:** (9) 927.
Templin MF, Stoll D, Schrenk M, Traub PC, Vohringer CF, Joos TO. 2002. Protein microarray technology (Review). *Drug Discov Today* **7:** (15) 815.
Tissot JD, Vu DH, Aubert V, Schneider P, Vuadens F, Crettaz D, Duchosal MA. 2002. The immunoglobulinopathies: From physiopathology to diagnosis - Review. *Proteomics* **2:** (7) 812.
Visca P, Leoni L, Wilson MJ, Lamont IL. 2002. Iron transport and regulation, cell signalling and genomics: Lessons from *Escherichia coli* and *Pseudomonas* (Microreview). *Mol Microbiol* **45:** (5) 1177.
Yang YH, Speed T. 2002. Design issues for cDNA microarray experiments. *Nat Rev Genet* **3:** (8) 579.
Yarmush ML, Jayaraman A. 2002. Advances in proteomic technologies. *Annu Rev Biomed Eng* **4:** 349.

### 3 Large-scale sequencing and mapping

Aparicio S, Chapman J, Stupka E, Putnam N, Chia J, Dehal P, Christoffels A, Rash S, Hoon S, Smit A *et al*. 2002. Whole-genome shotgun assembly and analysis of the genome of *Fugu rubripes*. *Science* **297:** (5585) 1301.
Brugmans B, Del Carmen AF, Bachem CWB, Van Os H, Van Eck HJ, Visser RGF. 2002. A novel method for the construction of genome wide transcriptome maps. *Plant J* **31:** (2) 211.
Chen LYY, Lu SH, Shih ESC, Hwang MJ. 2002. Single nucleotide polymorphism mapping using genome-wide unique sequences. *Genome Res* **12:** (7) 1106.
Cowman AF, Crabb BS. 2002. The *Plasmodium falciparum* genome: A blueprint for erythrocyte invasion. *Science* **298:** (5591) 126.
Eisen JA, Nelson KE, Paulsen IT, Heidelberg JF, Wu M, Dodson RJ, Deboy R, Gwinn ML, Nelson WC, Haft DH *et al*. 2002. The complete genome sequence of *Chlorobium tepidum* TLS, a photosynthetic, anaerobic, green-sulfur bacterium. *Proc Natl Acad Sci U S A* **99:** (14) 9509.
Gardner MJ, Hall N, Fung E, White O, Berriman M, Hyman RW, Carlton JM, Pain A, Nelson KE, Bowman S *et al*. 2002. Genome sequence of the human malaria parasite *Plasmodium falciparum*. *Nature* **419:** (6906) 498.
Gregory SG, Sekhon M, Schein J, Zhao SY, Osoegawa K, Scott CE, Evans RS, Burridge PW, Cox TV, Fox CA *et al*. 2002. A physical map of the mouse genome. *Nature* **418:** (6899) 743.
Holt RA, Subramanian GM, Halpern A, Sutton GG, Charlab R, Nusskern DR, Wincker P, Clark AG, Ribeiro JMC, Wides R *et al*. 2002. The genome sequence of the malaria mosquito *Anopheles gambiae*. *Science* **298:** (5591) 129.
Kutsenko AS, Gizatullin RZ, AlAmin AN, Wang FL, Kvasha SM,

In order to keep subscribers up-to-date with the latest developments in their field, this current awareness service is provided by John Wiley & Sons and contains newly-published material on comparative and functional genomics. Each bibliography is divided into 16 sections. 1 Reviews & symposia; 2 General; 3 Large-scale sequencing and mapping; 4 Evolutionary genomics; 5 Comparative genomics; 6 Pathways, gene families and regulons; 7 Pharmacogenomics; 8 EST, cDNA and other clone resources; 9 Functional genomics; 10 Transcriptomics; 11 Proteomics; 12 Protein structural genomics; 13 Metabolomics; 14 Genomic approaches to development; 15 Technological advances; 16 Bioinformatics. Within each section, articles are listed in alphabetical order with respect to author. If, in the preceding period, no publications are located relevant to any one of these headings, that section will be omitted.

Copyright © 2003 John Wiley & Sons, Ltd.

Podowski RM, Matushkin YG, Gyanchandani A, Muravenko OV, Levitsky VG *et al*. 2002. *Not*I flanking sequences: A tool for gene discovery and verification of the human genome. *Nucleic Acids Res* **30:** (14) 3163.

Swan KA, Curtis DE, McKusick KB, Voinov AV, Mapa FA, Cancilla MR. 2002. High-throughput gene mapping in *Caenorhabditis elegans*. *Genome Res* **12:** (7) 1100.

Xiao M, Zhu ZZ, Liu JP, Zhang CY. 2002. A new method based on entropy theory for genomic sequence analysis. *Acta Biotheoretica* **50:** (3) 155.

### 4 Evolutionary genomics

Bao ZR, Eddy SR. 2002. Automated *de novo* identification of repeat sequence families in sequenced genomes. *Genome Res* **12:** (8) 1269.

Daubin V, Gouy M, Perriere G. 2002. A phylogenomic approach to bacterial phylogeny: Evidence of a core of genes sharing a common history. *Genome Res* **12:** (7) 1080.

Devos KM, Brown JKM, Bennetzen JL. 2002. Genome size reduction through illegitimate recombination counteracts genome expansion in *Arabidopsis*. *Genome Res* **12:** (7) 1075.

Eisen JA, Wu M. 2002. Phylogenetic analysis and gene functional predictions: Phylogenomics in action. *Theor Popul Biol* **61:** (4) 481.

Jain R, Rivera MC, Moore JE, Lake JA. 2002. Horizontal gene transfer in microbial genome evolution. *Theor Popul Biol* **61:** (4) 489.

Karlin S, Brocchieri L, Trent J, Blaisdell BE, Mrazek J. 2002. Heterogeneity of genome and proteome content in bacteria, archaea, and eukaryotes. *Theor Popul Biol* **61:** (4) 367.

Lespinet O, Wolf YI, Koonin EV, Aravind L. 2002. The role of lineage-specific gene family expansion in the evolution of eukaryotes. *Genome Res* **12:** (7) 1048.

Mamedov I, Batrak A, Buzdin A, Arzumanyan E, Lebedev Y, Sverdlov ED. 2002. Genome-wide comparison of differences in the integration sites of interspersed repeats between closely related genomes (Online paper). *Nucleic Acids Res* **30:** (14) e71.

### 5 Comparative genomics

Bogdanov YF, Dadashev SY, Grishaeva TM. 2002. Comparative genomics and proteomics of *Drosophila*, Brenner's nematode, and *Arabidopsis*: Identification of functionally similar genes and proteins of meiotic chromosome synapsis. *Russ J Genet* **38:** (8) 908.

Carlton JM, Angiuoli SV, Suh BB, Kooij TW, Pertea M, Silva JC, Ermolaeva MD, Allen JE, Selengut JD, Koo HL *et al*. 2002. Genome sequence and comparative analysis of the model rodent malaria parasite *Plasmodium yoelii yoelii*. *Nature* **419:** (6906) 512.

Cole ST. 2002. Comparative mycobacterial genomics as a tool for drug target and antigen discovery. *Eur Resp J* **20:** (Suppl 36) S78.

Fulton TM, Van der Hoeven R, Eannetta NT, Tanksley SD. 2002. Identification, analysis, and utilization of conserved ortholog set markers for comparative genomics in higher plants. *Plant Cell* **14:** (7) 1457.

Iandolo JJ, Worrell V, Groicher KH, Qian YD, Tian RY, Kenton S, Dorman A, Ji HG, Lin SP, Loh P, Qi S, Zhu H, Roe BA. 2002. Comparative analysis of the genomes of the temperate bacteriophages $\phi$11, $\phi$12 and $\phi$13 of *Staphylococcus aureus* 8325. *Gene* **289:** (1-2) 109.

Jiang ZH, Melville JS, Cao HH, Kumar S, Filipski A, Gibbins AMV. 2002. Measuring conservation of contiguous sets of autosomal markers on bovine and porcine genomes in relation to the map of the human genome. *Genome* **45:** (4) 769.

Kaufman TC, Severson DW, Robinson GE. 2002. The *Anopheles* genome and comparative insect genomics. *Science* **298:** (5591) 97.

Zdobnov EM, Von Mering C, Letunic I, Torrents D, Suyama M, Copley RR, Christophides GK, Thomasova D, Holt RA, Subramanian GM *et al*. 2002. Comparative genome and proteome analysis of *Anopheles gambiae* and *Drosophila melanogaster*. *Science* **298:** (5591) 149.

### 6 Pathways, gene families and regulons

Christophides GK, Zdobnov E, Barillas-Mury C, Birney E, Blandin S, Blass C, Brey PT, Collins FH, Danielli A, Dimopoulos G *et al*. 2002. Immunity-related genes and gene families in *Anopheles gambiae*. *Science* **298:** (5591) 159.

De Haan G, Bystrykh LV, Weersing E, Dontje B, Geiger H, Ivanova N, Lemischka IR, Vellenga E, Van Zant G. 2002. A genetic and genomic analysis identifies a cluster of genes associated with hematopoietic cell turnover. *Blood* **100:** (6) 2056.

Forsyth MH, Cao P, Garcia PP, Hall JD, Cover TL. 2002. Genome-wide transcriptional profiling in a histidine kinase mutant of *Helicobacter pylori* identifies members of a regulon. *J Bacteriol* **184:** (16) 4630.

Halfon MS, Grad Y, Church GM, Michelson AM. 2002. Computation-based discovery of related transcriptional regulatory modules and motifs using an experimentally validated combinatorial model. *Genome Res* **12:** (7) 1019.

Remaley AT, Bark S, Walts AD, Freeman L, Shulenin S, Annilo T, Elgin E, Rhodes HE, Joyce C, Dean M *et al*. 2002. Comparative genome analysis of potential regulatory elements in the ABCG5-ABCG8 gene cluster. *Biochem Biophys Res Commun* **295:** (2) 276.

Rodriguez-Navarro S, Llorente B, Rodriguez-Manzaneque MT, Ramne A, Uber G, Marchesan D, Dujon B, Herrero E, Sunnerhagen P, Perez-Ortin JE. 2002. Functional analysis of yeast gene families involved in metabolism of vitamins $B_1$ and $B_6$. *Yeast* **19:** (14) 1261.

Zheng Y, Szustakowski JD, Fortnow L, Roberts RJ, Kasif S. 2002. Computational identification of operons in microbial genomes. *Genome Res* **12:** (8) 1221.

### 7 Pharmacogenomics

Alevizos I, Blaeser B, Gallagher G, Ohyama H, Wong DTW, Todd R. 2002. Odontogenic carcinoma: A functional genomic comparison with oral mucosal squamous cell carcinoma. *Oral Oncol* **38:** (5) 504.

Carroll JM, McElwee KJ, King LE, Byrne MC, Sundberg JP. 2002. Gene array profiling and immunomodulation studies define a cell-mediated immune response underlying the pathogenesis of alopecia areata in a mouse model and humans. *J Investig Dermatol* **119:** (2) 392.

Chalker AF, Lunsford RD. 2002. Rational identification of new antibacterial drug targets that are essential for viability using a genomics-based approach. *Pharmacol Ther* **95:** (1) 1.

Chen GA, Gharib TG, Huang CC, Thomas DG, Shedden KA, Taylor JMG, Kardia SLR, Misek DE, Giordano TJ, Iannettoni MD, Orringer MB, Hanash SM, Beer DG. 2002. Proteomic analysis of lung adenocarcinoma: Identification of a highly expressed set of proteins in tumors. *Clin Cancer Res* **8:** (7) 2298.

Chen ST, Pan LT, Tsai YC, Huang CM. 2002. Proteomics reveals protein profile changes in doxorubicin: Treated MCF-7 human breast cancer cells. *Cancer Lett* **181:** (1) 95.

Claudio JO, Masih-Khan E, Tang HC, Goncalves J, Voralia M, Li ZH, Nadeem V, Cukerman E, Francisco-Pabalan O, Liew CC, Woodgett JR, Stewart AK. 2002. A molecular compendium of genes expressed in multiple myeloma. *Blood* **100:** (6) 2175.

Cowen LE, Nantel A, Whiteway MS, Thomas DY, Tessier DC, Kohn LM, Anderson JB. 2002. Population genomics of drug resistance in *Candida albicans*. *Proc Natl Acad Sci U S A* **99:** (14) 9284.

De Longueville F, Surry D, Meneses-Lorente G, Bertholet V, Talbot V, Evrard S, Chandelier N, Pike A, Worboys P, Rasson JP *et al*. 2002. Gene expression profiling of drug metabolism and toxicology markers using a low-density DNA microarray. *Biochem Pharmacol* **64:** (1) 137.

Diehn M, Alizadeh AA, Rando OJ, Liu CL, Stankunas K, Botstein D, Crabtree GR, Brown PO. 2002. Genomic expression programs and the integration of the CD28 costimulatory signal in T-cell activation. *Proc Natl Acad Sci U S A* **99:** (18) 11796.

Fillmore GC, Lin ZS, Bohling SD, Robetorye RS, Kim CH, Jenson SD, Elenitoba-Johnson KSJ, Lim MS. 2002. Gene expression profiling of cell lines derived from T-cell malignancies. *FEBS Lett* **522:** (1-3) 183.

Gharib TG, Chen GA, Wang H, Huang CC, Prescott MS, Shedden K, Misek DE, Thomas DG, Giordano TJ, Taylor JMG *et al*. 2002. Proteomic analysis of cytokeratin isoforms uncovers association with survival in lung adenocarcinoma. *Neoplasia* **4:** (5) 440.

Gordon GJ, Jensen RV, Hsiao LL, Gullans SR, Blumenstock JE, Ramaswamy S, Richards WG, Sugarbaker DJ, Bueno R. 2002. Translation of microarray data into clinically relevant cancer diagnostic tests using gene expression ratios in lung cancer and mesothelioma. *Cancer Res* **62:** (17) 4963.

Grifantini R, Bartolini E, Muzzi A, Draghi M, Frigimelica E, Berger J, Ratti G, Petracca R, Galli G, Agnusdei M *et al*. 2002. Previously un-

Copyright © 2003 John Wiley & Sons, Ltd.

recognized vaccine candidates against group B meningococcus identified by DNA microarrays. *Nat Biotechnol* **20:** (9) 914.
Ha GH, Lee SU, Kang DG, Ha NY, Kim SH, Kim J, Bae JM, Kim JW, Lee CW. 2002. Proteome analysis of human stomach tissue: Separation of soluble proteins by two-dimensional polyacrylamide gel electrophoresis and identification by mass spectrometry. *Electrophoresis* **23:** (15) 2513.
Hartmann KA, Modlich O, Prisack HB, Gerlach B, Bojar H. 2002. Gene expression profiling of advanced head and neck squamous cell carcinomas and two squamous cell carcinoma cell lines under radio/chemotherapy using cDNA arrays. *Radiother Oncol* **63:** (3) 309.
Havlasova J, Hernychova L, Halada P, Pellantova W, Krejsek J, Stulik J, Macela A, Jungblut PR, Larsson P, Forsman M. 2002. Mapping of immunoreactive antigens of *Francisella tularensis* live vaccine strain. *Proteomics* **2:** (7) 857.
Hemby SE, Ginsberg SD, Brunk B, Arnold SE, Trojanowski JQ, Eberwine JH. 2002. Gene expression profile for schizophrenia: Discrete neuron transcription patterns in the entorhinal cortex. *Arch Gen Psychiatry* **59:** (7) 631.
Hwang JJ, Allen PD, Tseng GC, Lam CW, Fananapazir L, Dzau VJ, Liew CC. 2002. Microarray gene expression profiles in dilated and hypertrophic cardiomyopathic end-stage heart failure. *Physiol Genomics* **10:** (1) 31.
Imam-Sghiouar N, Laude-Lemaire I, Labas V, Pflieger D, Le Caer JP, Caron M, Nabias DK, Joubert-Caron R. 2002. Subproteomics analysis of phosphorylated proteins: Application to the study of B-lymphoblasts from a patient with Scott syndrome. *Proteomics* **2:** (7) 828.
Jazaeri AA, Yee CJ, Sotiriou C, Brantley KR, Boyd J, Liu ET. 2002. Gene expression profiles, of BRCA1-linked, BRCA2-linked, and sporadic ovarian cancers. *J Nat Cancer Inst* **94:** (13) 990.
Jessani N, Liu YS, Humphrey M, Cravatt BF. 2002. Enzyme activity profiles of the secreted and membrane proteome that depict cancer cell invasiveness. *Proc Natl Acad Sci U S A* **99:** (16) 10335.
Ji JF, Chen X, Leung SY, Chi JTA, Chu KM, Yuen ST, Li R, Chan ASY, Li JY, Dunphy N, So S. 2002. Comprehensive analysis of the gene expression profiles in human gastric cancer cell lines. *Oncogene* **21:** (42) 6549.
Juan HF, Lin JYC, Chang WH, Wu CY, Pan TL, Tseng MJ, Khoo KH, Chen ST. 2002. Biomic study of human myeloid leukemia cells differentiation to macrophages using DNA array, proteomic, and bioinformatic analytical methods. *Electrophoresis* **23:** (15) 2490.
Katsuma S, Shiojima S, Hirasawa A, Takagaki K, Kaminishi Y, Koba M, Hagidai Y, Murai M, Ohgi T, Yano J, Tsujimoto G. 2002. Global analysis of differentially expressed genes during progression of calcium oxalate nephrolithiasis. *Biochem Biophys Res Commun* **296:** (3) 544.
Kitahara O, Katagiri T, Tsunoda T, Harima Y, Nakamura Y. 2002. Classification of sensitivity or resistance of cervical cancers to ionizing radiation according to expression profiles of 62 genes selected by cDNA microarray analysis. *Neoplasia* **4:** (4) 295.
Lee S, Baek M, Kim HY, Ha JH, Jeoung DI. 2002. Mechanism of doxorubicin-induced cell death and expression profile analysis. *Biotechnol Lett* **24:** (14) 1147.
Li JN, Zhang Z, Rosenzweig J, Wang YY, Chan DW. 2002. Proteomics and bioinformatics approaches for identification of serum biomarkers to detect breast cancer. *Clin Chem* **48:** (8) 1296.
Li Y, Li YL, Tang R, Xu H, Qiu MY, Chen Q, Chen JX, Fu ZR, Ying K, Xie Y, Mao Y. 2002. Discovery and analysis of hepatocellular carcinoma genes using cDNA microarrays. *J Cancer Res Clin Oncol* **128:** (7) 369.
Luo LY, Herrera I, Soosaipillai A, Diamandis EP. 2002. Identification of heat shock protein 90 and other proteins as tumour antigens by serological screening of an ovarian carcinoma expression library. *Br J Cancer* **87:** (3) 339.
McDonough JL, Neverova I, Van Eyk JE. 2002. Proteomic analysis of human biopsy samples by single two-dimensional electrophoresis: Coomassie, silver, mass spectrometry, and Western blotting. *Proteomics* **2:** (8) 978.
Park S, Lee M, Kim HY, Jeoung DI. 2002. Proteome analysis of 5-fluorouracil-resistance-associated protein in human gastric cancer cells. *Biotechnol Lett* **24:** (14) 1141.
Piquemal D, Commes T, Manchon L, Lejeune M, Ferraz C, Pugnere D, Demaille J, Elalouf JM, Marti J. 2002. Transcriptome analysis of monocytic leukemia cell differentiation. *Genomics* **80:** (3) 361.
Pociot F, Karlsen AE. 2002. Combined genome and proteome approach to identify new susceptibility genes. *Am J Med Genet* **115:** (1) 55.
Pucci-Minafra I, Fontana S, Cancemi P, Basirico L, Caricato S, Minafra S. 2002. A contribution to breast cancer cell proteomics: Detection of new sequences. *Proteomics* **2:** (7) 919.
Rhodes DR, Barrette TR, Rubin MA, Ghosh D, Chinnaiyan AM. 2002. Meta-analysis of microarrays: Interstudy validation of gene expression profiles reveals pathway dysregulation in prostate cancer. *Cancer Res* **62:** (15) 4427.
Schwertz H, Langin T, Platsch H, Richert J, Bomm S, Schmidt M, Hillen H, Blaschke G, Meyer J, Darius H, Buerke M. 2002. Two-dimensional analysis of myocardial protein 2 expression following myocardial ischemia and reperfusion in rabbits. *Proteomics* **2:** (8) 988.
Watson MA, Gutmann DH, Peterson K, Chicoine MR, Kleinschmidt-de Masters BK, Brown HG, Perry A. 2002. Molecular characterization of human meningiomas by gene expression profiling using high-density oligonucleotide microarrays. *Am J Pathol* **161:** (2) 665.
Wikman H, Kettunen E, Seppanen JK, Karjalainen A, Hollmen J, Anttila S, Knuutila S. 2002. Identification of differentially expressed genes in pulmonary adenocarcinoma by using cDNA array. *Oncogene* **21:** (37) 5804.
Yano N, Fadden-Paiva KJ, Endoh M, Sakai H, Kurokawa K, Dworkin LD, Rifai A. 2002. Profiling the IgA nephropathy renal transcriptome: Analysis by complementary DNA array hybridization. *Nephrology* **7:** (Aug) S140.
Yao RS, Wang Y, Lubet RA, You M. 2002. Differentially expressed genes associated with mouse lung tumor progression. *Oncogene* **21:** (37) 5814.
Zhang HQ, Lu H, Enosawa S, Takahara S, Sakamoto K, Nakajima T, Saito H, Suzuki S. 2002. Microarray analysis of gene expression in peripheral blood mononuclear cells derived from long-surviving renal recipients. *Transplant Proc* **34:** (5) 1757.

### 8 EST, cDNA and other clone resources

Bailey J, Manoil C. 2002. Genome-wide internal tagging of bacterial exported proteins. *Nat Biotechnol* **20:** (8) 839.
Fahrenkrug SC, Smith TPL, Freking BA, Cho J, White J, Vallet J, Wise T, Rohrer G, Pertea E, Sultana R, Quackenbush J, Keele JW. 2002. Porcine gene discovery by normalized cDNA-library sequencing and EST cluster assembly. *Mamm Genome* **13:** (8) 475.
Inaba K, Padma P, Satouh Y, Shinl T, Kohara Y, Satoh N, Satou Y. 2002. EST analysis of gene expression in testis of the ascidian *Ciona intestinalis*. *Mol Reprod Dev* **62:** (4) 431.
Koumproglou R, Wilkes TM, Townson P, Wang XY, Beynon J, Pooni HS, Newbury HJ, Kearsey MJ. 2002. STAIRS: A new genetic resource for functional genomic studies of *Arabidopsis*. *Plant J* **31:** (3) 355.
Sonstegard TS, Capuco AV, White J, Van Tassell CP, Connor EE, Cho J, Sultana R, Shade L, Wray JE, Wells KD, Quackenbush J. 2002. Analysis of bovine mammary gland EST and functional annotation of the *Bos taurus* gene index. *Mamm Genome* **13:** (7) 373.
Van der Hoeven R, Ronning C, Giovannoni J, Martin G, Tanksley S. 2002. Deductions about the number, organization, and evolution of genes in the tomato genome based on analysis of a large expressed sequence tag collection and selective genomic sequencing. *Plant Cell* **14:** (7) 1441.

### 9 Functional genomics

Band MR, Olmstead C, Everts RE, Liu ZL, Lewin HA. 2002. A 3800 gene microarray for cattle functional genomics: Comparison of gene expression in spleen, placenta, and brain. *Anim Biotechnol* **13:** (1) 163.
Giaever G, Chu AM, Ni L, Connelly C, Riles L, Veronneau S, Dow S, Lucau-Danila A *et al*. 2002. Functional profiling of the *Saccharomyces cerevisiae* genome. *Nature* **418:** (6896) 387.
Lenaers G, Pelloquin L, Olichon A, Emorine LJ, Guillou E, Delettre C, Hamel CP, Ducommun B, Belenguer P. 2002. What similarity between human and fission yeast proteins is required for orthology? *Yeast* **19:** (13) 1125.
Peterson KA, King BL, Hagge-Greenberg A, Roix JJ, Bult CJ, O'Brien TP. 2002. Functional and comparative genomic analysis of the piebald

Copyright © 2003 John Wiley & Sons, Ltd. 

deletion region of mouse chromosome 14. *Genomics* **80:** (2) 172.
Scearce LM, Brestelli JE, McWeeney SK, Lee CS, Mazzarelli J, Pinney DF, Pizarro A, Stoeckert CJ, Clifton SW, Permutt MA, Brown J, Melton DA, Kaestner KH. 2002. Functional genomics of the endocrine pancreas: The pancreas clone set and PancChip, new resources for diabetes research. *Diabetes* **51:** (7) 1997.
Van der Leij FR, Cox KB, Jackson VN, Huijkman NCA, Bartelds B, Kuipers JRG, Dijkhuizen T, Terpstra P, Wood PA, Zammit VA, Price NT. 2002. Structural and functional genomics of the *CPT1B* gene for muscle-type carnitine palmitoyltransferase I in mammals. *J Biol Chem* **277:** (30) 26994.
Van Dijl JM, Braun PG, Robinson C, Quax WJ, Antelmann H, Hecker M, Muller J, Tjalsma H, Bron S, Jongbloed JDH. 2002. Functional genomic analysis of the *Bacillus subtilis* Tat pathway for protein secretion. *J Biotechnol* **98:** (2-3) 243.
Winssinger N, Ficarro S, Schultz PG, Harris JL. 2002. Profiling protein function with small molecule microarrays. *Proc Natl Acad Sci U S A* **99:** (17) 11139.
Wu LF, Hughes TR, Davierwala AP, Robinson MD, Stoughton R, Altschuler SJ. 2002. Large-scale prediction of *Saccharomyces cerevisiae* gene function using overlapping transcriptional clusters. *Nat Genet* **31:** (3) 255.
Zwiesler-Vollick J, Plovanich-Jones AE, Nomura K, Bandyopadhyay S, Joardar V, Kunkel BN, He SY. 2002. Identification of novel *hrp*-regulated genes through functional genomic analysis of the *Pseudomonas syringae* pv. tomato DC3000 genome. *Mol Microbiol* **45:** (5) 1207.

### I0 Transcriptomics

Ahn JH, Lee Y, Jeon C, Lee SJ, Lee BH, Choi KD, Bae YS. 2002. Identification of the genes differentially expressed in human dendritic cell subsets by cDNA subtraction and microarray analysis. *Blood* **100:** (5) 1742.
Aigner T, Finger F, Gebhard PM, Zien A, Zimmer R. 2002. Gene expression pattern of adult chondrocytes using cDNA-array technology (German, English Abstract). *Z Rheumatol* **61:** (3) 260.
Anisimov SV, Tarasov KV, Stern MD, Lakatta EG, Boheler KR. 2002. A quantitative and validated SAGE transcriptome reference for adult mouse heart. *Genomics* **80:** (2) 213.
Auger S, Denchin A, Martin-Verstraete I. 2002. Global expression profile of *Bacillus subtilis* grown in the presence of sulfate or methionine. *J Bacteriol* **184:** (18) 5179.
Beliaev AS, Thompson DK, Fields MW, Wu LY, Lies DP, Nealson KH, Zhou JZ. 2002. Microarray transcription profiling of a *Shewanella oneidensis etrA* mutant. *J Bacteriol* **184:** (16) 4612.
Bodyak N, Kang PM, Hiromura M, Sulijoadikusumo I, Horikoshi N, Khrapko K, Usheva A. 2002. Gene expression profiling of the aging mouse cardiac myocytes. *Nucleic Acids Res* **30:** (17) 3788.
Cao ZY, Wu HK, Bruce A, Wollenberg K, Panjwani N. 2002. Detection of differentially expressed genes in healing mouse corneas, using cDNA microarrays. *Invest Ophthalmol Vis Sci* **43:** (9) 2897.
Carson DD, Lagow E, Thathiah A, Al Shami R, Farach-Carson MC, Vernon M, Yuan LW, Fritz MA, Lessey B. 2002. Changes in gene expression during the early to mid-luteal (receptive phase) transition in human endometrium detected by high-density microarray screening. *Mol Hum Reprod* **8:** (9) 871.
D'Agata V, Warren ST, Zhao WQ, Torre ER, Alkon DL, Cavallaro S. 2002. Gene expression profiles in a transgenic animal model of fragile X syndrome. *Neurobiol Dis* **10:** (3) 211.
De Boer JM, McDermott JP, Wang XH, Maier T, Qu F, Hussey RS, Davis EL, Baum TJ. 2002. The use of DNA microarrays for the developmental expression analysis of cDNAs from the oesophageal gland cell region of *Heterodera glycines*. *Mol Plant Pathol* **3:** (4) 261.
Evans SJ, Datson NA, Kabbaj M, Thompson RC, Vreugdenhil E, De Kloet ER, Watson SJ, Akil H. 2002. Evaluation of Affymetrix Gene Chip sensitivity in rat hippocampal tissue using SAGE analysis. *Eur J Neurosci* **16:** (3) 409.
Fan JS, Yang XL, Wang WG, Wood WH, Becker KG, Gorospe M. 2002. Global analysis of stress-regulated mRNA turnover by using cDNA arrays. *Proc Natl Acad Sci U S A* **99:** (16) 10611.
Fowler S, Thomashow MF. 2002. *Arabidopsis* transcriptome profiling indicates that multiple regulatory pathways are activated during cold acclimation in addition to the CBF cold response pathway. *Plant Cell* **14:** (8) 1675.
Garcia T, Roman-Roman S, Jackson A, Theilhaber J, Connolly T, Spinella-Jaegle S, Kawai S, Courtois B, Bushnell S, Auberval M, Call K, Baron R. 2002. Behavior of osteoblast, adipocyte, and myoblast markers in genome-wide expression analysis of mouse calvaria primary osteoblasts *in vitro*. *Bone* **31:** (1) 205.
Guo YLL, Chang HC, Tsai JH, Huang JC, Li C, Young KC, Wu LW, Lai MD, Liu HS, Huang WY. 2002. Two UVC-induced stress response pathways in HeLa cells identified by cDNA microarray. *Environ Mol Mutagen* **40:** (2) 122.
Hayashi M, Mizoguchi H, Shiraishi N, Obayashi M, Nakagawa S, Imai J, Watanabe S, Ota T, Ikeda M. 2002. Transcriptome analysis of acetate metabolism in *Corynebacterium glutamicum* using a newly developed metabolic array. *Biosci Biotechnol Biochem* **66:** (6) 1337.
Kita H, Carmichael J, Swartz J, Muro S, Wyttenbach A, Matsubara K, Rubinsztein DC, Kato K. 2002. Modulation of polyglutamine-induced cell death by genes identified by expression profiling. *Hum Mol Genet* **11:** (19) 2279.
Kitagawa E, Takahashi J, Momose Y, Iwahashi H. 2002. Effects of the pesticide thiuram: Genome-wide screening of indicator genes by yeast DNA microarray. *Environ Sci Technol* **36:** (18) 3908.
Kubo T, Yamashita T, Yamaguchi A, Hosokawa K, Tohyama M. 2002. Analysis of genes induced in peripheral nerve after axotomy using cDNA microarrays. *J Neurochem* **82:** (5) 1129.
Levsky JM, Shenoy SM, Pezo RC, Singer RH. 2002. Single-cell gene expression profiling. *Science* **297:** (5582) 836.
Lombardia LJ, Becerra M, Rodriguez-Belmonte E, Hauser NC, Cerdan ME. 2002. Genome-wide analysis of yeast transcription upon calcium shortage. *Cell* **32:** (2) 83.
Mader U, Homuth G, Scharf C, Buttner K, Bode R, Hecker M. 2002. Transcriptome and proteome analysis of *Bacillus subtilis* gene expression modulated by amino acid availability. *J Bacteriol* **184:** (15) 4288.
Maurer MH, Frietsch T, Waschke KF, Kuschinsky W, Gassmann M, Schneider A. 2002. Cerebral transcriptome analysis of transgenic mice overexpressing erythropoietin. *Neurosci Lett* **327:** (3) 181.
Misra J, Schmitt W, Hwang D, Hsiao LL, Gullans S, Stephanopoulos G, Stephanopoulos G. 2002. Interactive exploration of microarray gene expression patterns in a reduced dimensional space. *Genome Res* **12:** (7) 1112.
Muffler A, Bettermann S, Haushalter M, Horlein A, Neveling U, Schramm M, Sorgenfrei O. 2002. Genome-wide transcription profiling of *Corynebacterium glutamicum* after heat shock and during growth on acetate and glucose. *J Biotechnol* **98:** (2-3) 255.
Okinaka Y, Yang CH, Perna NT, Keen NT. 2002. Microarray profiling of *Erwinia chrysanthemi* 3937 genes that are regulated during plant infection. *Mol Plant Microbe Interact* **15:** (7) 619.
Okuda T, Sumiya T, Iwai N, Miyata T. 2002. Difference of gene expression profiles in spontaneous hypertensive rats and Wistar-Kyoto rats from two sources. *Biochem Biophys Res Commun* **296:** (3) 537.
Oshima T, Wada C, Kawagoe Y, Ara T, Maeda M, Masuda Y, Hiraga S, Mori H. 2002. Genome-wide analysis of deoxyadenosine methyltransferase-mediated control of gene expression in *Escherichia coli*. *Mol Microbiol* **45:** (3) 673.
Roth SM, Ferrell RE, Peters DG, Metter EJ, Hurley BF, Rogers MA. 2002. Influence of age, sex, and strength training on human muscle gene expression determined by microarray. *Physiol Genomics* **10:** (3) 181.
Seki M, Narusaka M, Ishida J, Nanjo T, Fujita M, Oono Y, Kamiya A, Nakajima M, Enju A, Sakurai T *et al*. 2002. Monitoring the expression profiles of 7000 *Arabidopsis* genes under drought, cold and high-salinity stresses using a full-length cDNA microarray. *Plant J* **31:** (3) 279.
Sipione S, Rigamonti D, Valenza M, Zuccato C, Conti L, Pritchard J, Kooperberg C, Olson JM, Cattaneo E. 2002. Early transcriptional profiles in huntingtin-inducible striatal cells by microarray analyses. *Hum Mol Genet* **11:** (17) 1953.
Sironen RK, Karjalainen HM, Elo MA, Kaarniranta K, Torronen K, Takigawa M, Helminen HJ, Lammi MJ. 2002. cDNA array reveals mechanosensitive genes in chondrocytic cells under hydrostatic pressure. *Biochim Biophys Acta* **1591:** (1-3) 45.
Smith JJ, Marelli M, Christmas RH, Vizeacoumar FJ, Dilworth DJ, Ideker T, Galitski T, Dimitrov K, Rachubinski RA, Aitchison JD. 2002. Transcriptome profiling to identify genes involved in peroxisome assembly and function. *J Cell Biol* **158:** (2) 259.
Thomas SW, Glaring MA, Rasmussen SW, Kinane JT, Oliver RP. 2002.

Copyright © 2003 John Wiley & Sons, Ltd. 

Transcript profiling in the barley mildew pathogen *Blumeria graminis* by serial analysis of gene expression (SAGE). *Mol Plant Microbe Interact* **15:** (8) 847.
Tjaden B, Saxena RM, Stolyar S, Haynor DR, Kolker E, Rosenow C. 2002. Transcriptome analysis of *Escherichia coli* using high-density oligonucleotide probe arrays. *Nucleic Acids Res* **30:** (17) 3732.
Tsangaris GT, Botsonis A, Politis I, Tzortzatou-Stathopoulou F. 2002. Evaluation of cadmium-induced transcriptome alterations by three color cDNA labeling microarray analysis on a T-cell line. *Toxicology* **178:** (2) 135.
Watanabe J, Sasaki M, Suzuki Y, Sugano S. 2002. Analysis of transcriptomes of human malaria parasite *Plasmodium falciparum* using full-length enriched library: identification of novel genes and diverse transcription start sites of messenger RNAs. *Gene* **291:** (1-2) 105.
Weber A, Jung K. 2002. Profiling early osmostress-dependent gene expression in *Escherichia coli* using DNA microarrays. *J Bacteriol* **184:** (19) 5502.
Weindruch R, Kayo T, Lee CK, Prolla TA. 2002. Gene expression profiling of aging using DNA microarrays. *Mech Ageing Dev* **123:** (2-3) 177.
Weston GC, Haviv I, Rogers PAW. 2002. Microarray analysis of VEGF-responsive genes in myometrial endothelial cells. *Mol Hum Reprod* **8:** (9) 855.
Xu Q, Modrek B, Lee C. 2002. Genome-wide detection of tissue-specific alternative splicing in the human transcriptome. *Nucleic Acids Res* **30:** (17) 3754.
Yajima N, Masuda M, Miyazaki M, Nakajima N, Chien S, Shyy JYJ. 2002. Oxidative stress is involved in the development of experimental abdominal aortic aneurysm: A study of the transcription profile with complementary DNA microarray. *J Vasc Surg* **36:** (2) 379.
Yoshida S, Yashar BM, Hiriyanna S, Swaroop A. 2002. Microarray analysis of gene expression in the aging human retina. *Invest Ophthalmol Vis Sci* **43:** (8) 2554.
Zhou J, Jin Y, Gao Y, Wang H, Hu G, Huang Y, Chen Q, Feng M, Wu C. 2002. Genomic-scale analysis of gene expression profiles in TNF-α treated human umbilical vein endothelial cells. *Inflamm Res* **51:** (7) 332.

### II Proteomics

Adam GC, Sorensen EJ, Cravatt BF. 2002. Proteomic profiling of mechanistically distinct enzyme classes using a common chemotype. *Nat Biotechnol* **20:** (8) 805.
Agarwal GK, Rakwal R, Yonekura M, Kubo A, Saji H. 2002. Proteome analysis of differentially displayed proteins as a tool for investigating ozone stress in rice (*Oryza sativa* L.) seedlings. *Proteomics* **2:** (8) 947.
Asirvatham VS, Watson BS, Sumner LW. 2002. Analytical and biological variances associated with proteomic studies of *Medicago truncatula* by two-dimensional polyacrylamide gel electrophoresis. *Proteomics* **2:** (8) 960.
Beyer K, Bardina L, Grishina G, Sampson HA. 2002. Identification of sesame seed allergens by 2-dimensional proteomics and Edman sequencing: Seed storage proteins as common food allergens. *J Allergy Clin Immunol* **110:** (1) 154.
Cavaletto M, Giuffrida MG, Fortunato D, Gardano L, Dellavalle G, Napolitano L, Giunta C, Bertino E, Fabris C, Conti A. 2002. A proteomic approach to evaluate the butyrophilin gene family expression in human milk fat globule membrane. *Proteomics* **2:** (7) 850.
Cronshaw JA, Krutchinsky AN, Zhang WZ, Chait BT, Matunis MJ. 2002. Proteomic analysis of the mammalian nuclear pore complex. *J Cell Biol* **158:** (5) 915.
El Fakhry Y, Ouellette M, Papadopoulou B. 2002. A proteomic approach to identify developmentally regulated proteins in *Leishmania infantum*. *Proteomics* **2:** (8) 1007.
Eschenbrenner M, Wagner MA, Horn TA, Kraycer JA, Mujer CV, Hagius S, Elzer P, Del Vecchio VG. 2002. Comparative proteome analysis of *Brucella melitensis* vaccine strain Rev 1 and a virulent strain, 16M. *J Bacteriol* **184:** (18) 4962.
Fessler MB, Malcolm KC, Duncan MW, Worthen GS. 2002. A genomic and proteomic analysis of activation of the human neutrophil by lipopolysaccharide and its mediation by p38 mitogen-activated protein kinase. *J Biol Chem* **277:** (35) 31291.
Florens L, Washburn MP, Raine JD, Anthony RM, Grainger M, Haynes JD, Moch JK, Muster N, Sacci JB, Tabb DL *et al*. 2002. A proteomic view of the *Plasmodium falciparum* life cycle. *Nature* **419:** (6906) 520.
Fukao Y, Hayashi M, Nishimura M. 2002. Proteomic analysis of leaf peroxisomal proteins in greening cotyledons of *Arabidopsis thaliana*. *Plant Cell Physiol* **43:** (7) 689.
Gupta S, Pandit SB, Srinivasan N, Chatterji D. 2002. Proteomics analysis of carbon-starved *Mycobacterium smegmatis*: Induction of Dps-like protein. *Protein Eng* **15:** (6) 503.
Jenkins LW, Peters GW, Dixon CE, Zhang X, Clark RSB, Skinner JC, Marion DW, Adelson PD, Kochanek PM. 2002. Conventional and functional proteomics using large format two-dimensional gel electrophoresis 24 hours after controlled cortical impact in postnatal day 17 rats. *J Neurotrauma* **19:** (6) 715.
Journet A, Chapel A, Kieffer S, Roux F, Garin J. 2002. Proteomic analysis of human lysosomes: Application to monocytic and breast cancer cells. *Proteomics* **2:** (8) 1026.
Koller A, Washburn MP, Lange BM, Andon NL, Deciu C, Haynes PA, Hays L, Schieltz D, Ulaszek R, Wei J *et al*. 2002. Proteomic survey of metabolic pathways in rice. *Proc Natl Acad Sci U S A* **99:** (18) 11969.
Lasonder E, Ishihama Y, Andersen JS, Vermunt AMW, Pain A, Sauerwein RW, Eling WMC, Hall N, Waters AP, Stunnenberg HG, Mann M. 2002. Analysis of the *Plasmodium falciparum* proteome by high-accuracy mass spectrometry. *Nature* **419:** (6906) 537.
Lipton MS, Pasa-Tolic L, Anderson GA, Anderson DJ, Auberry DL, Battista KR, Daly MJ, Fredrickson J, Hixson KK, Kostandarithes H *et al*. 2002. Global analysis of the *Deinococcus radiodurans* proteome by using accurate mass tags. *Proc Natl Acad Sci U S A* **99:** (17) 11049.
Misumi S, Fuchigami T, Takamune N, Takahashi I, Takama M, Shoji S. 2002. Three isoforms of cyclophilin A associated with human immunodeficiency virus type 1 were found by proteomics by using two-dimensional gel electrophoresis and matrix-assisted laser desorption/ionization-time of flight mass spectrometry. *J Virol* **76:** (19) 10000.
Molloy MP, Phadke ND, Chen H, Tyldesley R, Garfin DE, Maddock JR, Andrews PC. 2002. Profiling the alkaline membrane proteome of *Caulobacter crescentus* with two-dimensional electrophoresis and mass spectrometry. *Proteomics* **2:** (7) 899.
Rappsilber J, Ryder U, Lamond AI, Mann M. 2002. Large-scale proteomic analysis of the human splicesome. *Genome Res* **12:** (8) 1231.
Rashid KA, Hevi S, Chen Y, Le Caherec F, Chuck SL. 2002. A proteomic approach identifies proteins in hepatocytes that bind nascent apolipoprotein B. *J Biol Chem* **277:** (24) 22010.
Skynner HA, Rosahl TW, Knowles MR, Salim K, Reid L, Cothliff R, McAllister G, Guest PC. 2002. Alterations of stress related proteins in genetically altered mice revealed by two-dimensional differential in-gel electrophoresis analysis. *Proteomics* **2:** (8) 1018.
Thiede B, Siejak F, Dimmler C, Rudel T. 2002. Prediction of translocation and cleavage of heterogeneous ribonuclear proteins and Rho guanine nucleotide dissociation inhibitor 2 during apoptosis by subcellular proteome analysis. *Proteomics* **2:** (8) 996.
Wagner MA, Eschenbrenner M, Horn TA, Kraycer JA, Mujer CV, Hagius S, Elzer P, Del Vecchio VG. 2002. Global analysis of the *Brucella melitensis* proteome: Identification of proteins expressed in laboratory-grown culture. *Proteomics* **2:** (8) 1047.
Willemoes M, Kilstrup M, Roepstorff P, Hammer K. 2002. Proteome analysis of a *Lactococcus lactis* strain overexpressing *gapA* suggests that the gene product is an auxillary glyceraldehyde 3-phosphate dehydrogenase. *Proteomics* **2:** (8) 1041.
Zhou ZL, Licklider LJ, Gygi SP, Reed R. 2002. Comprehensive proteomic analysis of the human spliceosome. *Nature* **419:** (6903) 182.

### I2 Protein structural genomics

Lesley SA, Kuhn P, Godzik A, Deacon AM, Mathews I, Kreusch A, Spraggon G, Klock HE, McMullan D, Shin T *et al*. 2002. Structural genomics of the *Thermotoga maritima* proteome implemented in a high-throughput structure determination pipeline. *Proc Natl Acad Sci U S A* **99:** (18) 11664.
Mallick P, Boutz DR, Eisenberg D, Yeates TO. 2002. Genomic evidence that the intracellular proteins of archaeal microbes contain disulfide bonds. *Proc Natl Acad Sci U S A* **99:** (15) 9679.

Copyright © 2003 John Wiley & Sons, Ltd. 

Pedelacq JD, Piltch E, Liong EC, Berendzen J, Kim CY, Rho BS, Park MS, Terwilliger TC, Waldo GS. 2002. Engineering soluble proteins for structural genomics. *Nat Biotechnol* **20:** (9) 927.

### I3 Metabolomics

Forster J, Gombert AK, Nielsen J. 2002. A functional genomics approach using metabolomics and *in silico* pathway analysis. *Biotechnol Bioeng* **79:** (7) 703.

Grosu P, Townsend JP, Hartl DL, Cavalieri D. 2002. Pathway processor: A tool for integrating whole-genome expression results into metabolic networks. *Genome Res* **12:** (7) 1121.

Plumb RS, Stumpf CL, Gorenstein MV, Castro-Perez JM, Dear GJ, Anthony M, Sweatman BC, Connor SC, Haselden JN. 2002. Metabonomics: The use of electrospray mass spectrometry coupled to reversed-phase liquid chromatography shows potential for the screening of rat urine in drug development. *Rapid Commun Mass Spectrom* **16:** (20) 1991.

### I4 Genomic approaches to development

Bardel J, Louwagie M, Jaquinod M, Jourdain A, Luche S, Rabilloud T, Macherel D, Garin J, Bourguignon J. 2002. A survey of the plant mitochondrial proteome 2 in relation to development. *Proteomics* **2:** (7) 880.

Brambrink T, Wabnitz P, Halter R, Klocke R, Carnwath J, Kues W, Wrenzycki C, Pau D, Niemann H. 2002. Application of cDNA arrays to monitor mRNA profiles in single preimplantation mouse embryos. *Biotechniques* **33:** (2) 376.

Egger B, Leemans R, Loop T, Kammermeier L, Fan Y, Radimerski T, Strahm MC, Certa U, Reichert H. 2002. Gliogenesis in *Drosophila*: Genome-wide analysis of downstream genes of glial cells missing in the embryonic nervous system. *Development* **129:** (14) 3295.

Gerritsen ME, Soriano R, Yang S, Ingle G, Zlot C, Toy K, Winer J, Draksharapu A, Peale F, Wu TD, Williams PM. 2002. *In silico* data filtering to identify new angiogenesis targets from a large *in vitro* gene profiling data set. *Physiol Genomics* **10:** (1) 13.

Heikinheimo K, Jee KJ, Niini T, Aalto Y, Happonen RP, Leivo I, Knuutila S. 2002. Gene expression profiling of ameloblastoma and human tooth germ by means of a cDNA microarray. *J Dent Res* **81:** (8) 525.

Ma XY, Husain T, Peng H, Lin S, Mironenko O, Maun N, Johnson S, Tuck D, Berliner N, Krause DS, Perkins S. 2002. Development of a murine hematopoietic progenitor complementary DNA microarray using a subtracted complementary DNA library. *Blood* **100:** (3) 833.

Moran JL, Li YZ, Hill AA, Mounts WM, Miller CP. 2002. Gene expression changes during mouse skeletal myoblast differentiation revealed by transcriptional profiling. *Physiol Genomics* **10:** (2) 103.

Rho J, Altman CR, Socci ND, Merkov L, Kim N, So H, Lee O, Takami M, Brivanlou AH, Choi Y. 2002. Gene expression profiling of osteoclast differentiation by combined suppression subtractive hybridization (SSH) and cDNA microarray analysis. *DNA Cell Biol* **21:** (8) 541.

Rivolta MN, Halsall A, Johnson CM, Tones MA, Holley MC. 2002. Transcript profiling of functionally related groups of genes during conditional differentiation of a mammalian cochlear hair cell line. *Genome Res* **12:** (7) 1091.

Ruuska SA, Girke T, Benning C, Ohlrogge JB. 2002. Contrapuntal networks of gene expression during *Arabidopsis* seed filling. *Plant Cell* **14:** (6) 1191.

Simin K, Scuderi A, Reamey J, Dunn D, Weiss R, Metherall JE, Letsou A. 2002. Profiling patterned transcripts in *Drosophila* embryos. *Genome Res* **12:** (7) 1040.

Tan SS, Gunnersen J, Job C. 2002. Global gene expression analysis of developing neocortex using SAGE. *Int J Dev Biol* **46:** (4) 653.

Ton C, Stamatiou D, Dzau VJ, Liew CC. 2002. Construction of a zebrafish cDNA microarray: Gene expression profiling of the zebrafish during development. *Biochem Biophys Res Commun* **296:** (5) 1134.

Unda FJ, Iehara N, De Vega S, De la Fuente M, Vilaxa A, Cobourne M, Sharpe PT, Yamada Y. 2002. Analysis of cDNAs from a mouse embryo tooth library: Identification of novel genes during tooth development. *Connect Tissue Res* **43:** (2-3) 176.

Van Zyl L, Von Arnold S, Bozhkov P, Chen Y, Egertsdotter U, MacKay J, Sederoff R, Shen J, Zelena L, Clapham DH. 2002. Heterologous array analysis in Pinaceae: Hybridization of *Pinus taeda* cDNA arrays with cDNA from needles and embryogenic cultures of *P. taeda, P. sylvestris* or *Picea abies*. *Comp Funct Genom* **3:** (4) 306.

### I5 Technological advances

Abdi FA, Mundt M, Doggett N, Bradbury EM, Chen X. 2002. Validation of DNA sequences using mass spectrometry coupled with nucleoside mass tagging. *Genome Res* **12:** (7) 1135.

Acestor N, Masina S, Walker J, Saravia NG, Fasel N, Quadroni M. 2002. Establishing two-dimensional gels for the analysis of *Leishmania* proteomes. *Proteomics* **2:** (7) 877.

Bateman RH, Carruthers R, Hoyes JB, Jones C, Langridge JI, Millar A, Vissers JPC. 2002. A novel precursor ion discovery method on a hybrid quadrupole orthogonal acceleration time-of-flight (Q-TOF) mass spectrometer for studying protein phosphorylation. *J Am Soc Mass Spectrom* **13:** (7) 792.

Battersby BJ, Lawrie GA, Johnston APR, Trau M. 2002. Optical barcoding of colloidal suspensions: Applications in genomics, proteomics and drug discovery. *Chem Commun* (14) 1435.

Bienvenut WV, Deon C, Pasquarello C, Campbell JM, Sanchez JC, Vestal ML, Hochstrasser DF. 2002. Matrix-assisted laser desorption/ionization-tandem mass spectrometry with high resolution and sensitivity for identification and characterization of proteins. *Proteomics* **2:** (7) 868.

Bose AK, Ing YH, Lavlinskaia N, Sareen C, Pramanik BN, Bartner PL, Liu YH, Heimark L. 2002. Microwave enhanced Akabori reaction for peptide analysis. *J Am Soc Mass Spectrom* **13:** (7) 839.

Brookes PS, Pinner A, Ramachandran A, Coward L, Barnes S, Kim H, Darley-Usmar VM. 2002. High throughput two-dimensional blue-native electrophoresis: A tool for functional proteomics of mitochondria and signaling complexes. *Proteomics* **2:** (8) 969.

Craven RA, Jackson DH, Selby PJ, Banks RE. 2002. Increased protein entry together with improved focussing using a combined IPGphor/Multiphor approach. *Proteomics* **2:** (8) 1061.

Fryksdale BG, Jedrzejewski PT, Wong DL, Gaertner AL, Miller BS. 2002. Impact of deglycosylation methods on two-dimensional gel electrophoresis and matrix assisted laser desorption/ionization-time of flight-mass spectrometry for proteomic analysis. *Electrophoresis* **23:** (14) 2184.

Hering JA, Innocent PR, Haris PI. 2002. Automatic amide I frequency selection for rapid quantification of protein secondary structure from Fourier transform infrared spectra of proteins. *Proteomics* **2:** (7) 839.

Huang Y, Joo S, Duhon M, Heller M, Wallace B, Xu X. 2002. Dielectrophoretic cell separation and gene expression profiling on microelectronic chip arrays. *Anal Chem* **74:** (14) 3362.

Jenssen TK, Langaas M, Kuo WP, Smith-Sorensen B, Myklebost O, Hovig E. 2002. Analysis of repeatability in spotted cDNA microarrays. *Nucleic Acids Res* **30:** (14) 3235.

Lee H, Griffin TJ, Gygi SP, Rist B, Aebersold R. 2002. Development of a multiplexed microcapillary liquid chromatography system for high-throughput proteome analysis. *Anal Chem* **74:** (17) 4353.

Lesaicherre ML, Lue RYP, Chen GYJ, Zhu Q, Yao SQ. 2002. Intein-mediated biotinylation of proteins and its application in a protein microarray. *J Am Chem Soc* **124:** (30) 8768.

Loo RRO, Loo JA, Du P, Holler T. 2002. *In vivo* labeling: A glimpse of the dynamic proteome and additional constraints for protein identification. *J Am Soc Mass Spectrom* **13:** (7) 804.

Manduchi E, Scearce LM, Brestelli JE, Grant GR, Kaestner KH, Stoeckert CJ. 2002. Comparison of different labeling methods for two-channel high-density microarray experiments. *Physiol Genomics* **10:** (3) 169.

Panifilov O, Lanne B. 2002. Peptide mass fingerprinting from wet and dry two-dimensional gels and its application in proteomics. *Anal Biochem* **307:** (2) 393.

Renault JP, Bernard A, Juncker D, Michel B, Bosshard HR, Delamarche E. 2002. Fabricating microarrays of functional proteins using affinity contact printing. *Angew Chem Int Ed* **41:** (13) 2320.

Ross ARS, Lee PJ, Smith DL, Langridge JI, Whetton AD, Gaskell SJ. 2002. Identification of proteins from two-dimensional polyacrylamide gels using a novel acid-labile surfactant. *Proteomics* **2:** (7) 928.

Copyright © 2003 John Wiley & Sons, Ltd. 

Schlosser A, Bodem J, Bossemeyer D, Grummt I, Lehmann WD. 2002. Identification of protein phosphorylation sites by combination of elastase digestion, immobilized metal affinity chromatography, and quadrupole-time of flight tandem mass spectrometry. *Proteomics* **2:** (7) 911.

Shah HN, Keys CJ, Schmid O, Gharbia SE. 2002. Matrix-assisted laser desorption/ionization time-of-flight mass spectrometry and proteomics: A new era in anaerobic microbiology. *Clin Infect Dis* **35:** (Suppl 1) 58.

Shen YF, Zhao R, Berger SJ, Anderson GA, Rodriguez N, Smith RD. 2002. High-efficiency nanoscale liquid chromatography coupled on-line with mass spectrometry using nanoelectrospray ionization for proteomics. *Anal Chem* **74:** (16) 4235.

Vissers JPC, Blackburn RK, Moseley MA. 2002. A novel interface for variable flow nanoscale LC/MS/MS for improved proteome coverage. *J Am Soc Mass Spectrom* **13:** (7) 760.

Wattenberg A, Organ AJ, Schneider K, Tyldesley R, Bordoli R, Bateman RH. 2002. Sequence dependent fragmentation of peptides generated by MALDI quadrupole time-of-flight (MALDI Q-TOF) mass spectrometry and its implications for protein identification. *J Am Soc Mass Spectrom* **13:** (7) 772.

Xiang CC, Kozhich OA, Chen M, Inman JM, Phan QN, Chen YD, Brownstein MJ. 2002. Amine-modified random primers to label probes for DNA microarrays. *Nat Biotechnol* **20:** (7) 738.

Yergey AL, Coorssen JR, Backlund PS, Blank PS, Humphrey GA, Zimmerberg J, Campbell JM, Vestal ML. 2002. *De novo* sequencing of peptides using MALDI/TOF-TOF. *J Am Soc Mass Spectrom* **13:** (7) 784.

Zhang RJ, Sioma CS, Thompson RA, Xiong L, Regnier FE. 2002. Controlling deuterium isotope effects in comparative proteomics. *Anal Chem* **74:** (15) 3662.

### I6 Bioinformatics

Abascal F, Valencia A. 2002. Clustering of proximal sequence space for the identification of protein families. *Bioinformatics* **18:** (7) 908.

Aude JC, Louis A. 2002. An incremental algorithm for *Z*-value computations. *Comput Chem* **26:** (5) 402.

Baisnee PF, Hampson S, Baldi P. 2002. Why are complementary DNA strands symmetric? *Bioinformatics* **18:** (8) 1021.

Bo TH, Jonassen I, Eidhammer I, Helgesen C. 2002. A fast top-down method for constructing reliable radiation hybrid frameworks. *Bioinformatics* **18:** (1) 11.

Bober M, Wiehe K, Yung C, Suzek TO, Lin M, Baumgartner W, Winslow R. 2002. CaGE: Cardiac gene expression knowledgebase. *Bioinformatics* **18:** (7) 1013.

Bonfield JK, Staden R. 2002. ZTR: A new found format for DNA sequence trace data. *Bioinformatics* **18:** (1) 3.

Bonfield JK, Beal KF, Betts MJ, Staden R. 2002. Trev: A DNA trace editor and viewer. *Bioinformatics* **18:** (1) 194.

Broet P, Richardson S, Radvanyi F. 2002. Bayesian hierarchical model for identifying changes in gene expression from microarray experiments. *J Comput Biol* **9:** (4) 671.

Bronner G, Spataro B, Page M, Gautier C, Rechenmann F. 2002. Modeling comparative mapping using objects and associations. *Comput Chem* **26:** (5) 413.

Buzko O, Shokat KM. 2002. A kinase sequence database: Sequence alignments and family assignment. *Bioinformatics* **18:** (9) 1274.

Cannata N, Toppo S, Romualdi C, Valle G. 2002. Simplifying amino acid alphabets by means of a branch and bound algorithm and substitution matrices. *Bioinformatics* **18:** (8) 1102.

Chang BCH, Halgamuge SK. 2002. Protein motif extraction with neuro-fuzzy optimization. *Bioinformatics* **18:** (8) 1084.

Chapman S, Schenk P, Kazan K, Manners J. 2002. Using biplots to interpret gene expression patterns in plants. *Bioinformatics* **18:** (1) 202.

Chen S, Lesnik EA, Hall TA, Sampath R, Griffey RH, Ecker DJ, Blyn LB. 2002. A bioinformatics based approach to discover small RNA genes in the *Escherichia coli* genome. *Biosystems* **65:** (2-3) 157.

Chen YD, Kamat V, Dougherty ER, Bittner ML, Meltzer PS, Trent JM. 2002. Ratio statistics of gene expression levels and applications to microarray data analysis. *Bioinformatics* **18:** (9) 1207.

Chetouani F, Glaser P, Kunst F. 2002. DiffTool: Building, visualizing and querying protein clusters. *Bioinformatics* **18:** (8) 1143.

Clare A, King RD. 2002. Machine learning of functional class from phenotype data. *Bioinformatics* **18:** (1) 160.

Clark T, Lee S, Scott LR, Wang SM. 2002. Computational analysis of gene identification with SAGE. *J Comput Biol* **9:** (3) 513.

Collado-Vides J, Moreno-Hagelsieb G, Medrano-Soto A. 2002. Microbial computational genomics of gene regulation. *Pure Appl Chem* **74:** (6) 899.

Comet JP, Henry J. 2002. Pairwise sequence alignment using a PROSITE pattern-derived similarity score. *Comput Chem* **26:** (5) 421.

Conant GC, Wagner A. 2002. GenomeHistory: A software tool and its application to fully sequenced genomes. *Nucleic Acids Res* **30:** (15) 3378.

Conklin D, Jonassen I, Aasland R, Taylor WR. 2002. Association of nucleotide patterns with gene function classes: Application to human 3′ untranslated sequences. *Bioinformatics* **18:** (1) 182.

Contreras-Moreira B, Bates PA. 2002. Domain Fishing: A first step in protein comparative modelling. *Bioinformatics* **18:** (8) 1141.

Cotter PJ, Caffrey DR, Shields DC. 2002. Improved database searches for orthologous sequences by conditioning on outgroup sequences. *Bioinformatics* **18:** (1) 83.

DeGroot AS, Sbai H, Aubin CS, McMurry J, Martin W. 2002. Immuno-informatics: Mining genomes for vaccine components. *Immunol Cell Biol* **80:** (3) 255.

Demir E, Babur O, Dogrusoz U, Gursoy A, Nisanci G, Cetin-Atalay R, Ozturk M. 2002. PATIKA: An integrated visual environment for collaborative construction and analysis of cellular pathways. *Bioinformatics* **18:** (7) 996.

Dodson JM, Charles PT, Stenger DA, Pancrazio JJ. 2002. Quantitative assessment of filter-based cDNA microarrays: Gene expression profiles of human T-lymphoma cell lines. *Bioinformatics* **18:** (7) 953.

Fradera X, De la Cruz X, Silva CHTP, Gelpi JL, Luque FJ, Orozco M. 2002. Ligand-induced changes in the binding sites of proteins. *Bioinformatics* **18:** (7) 939.

Friedberg I, Margalit H. 2002. PeCoP: Automatic determination of persistently conserved positions in protein families. *Bioinformatics* **18:** (9) 1276.

Gadgil C, Rink A, Beattie C, Hu WS. 2002. A mathematical model for suppression subtractive hybridization. *Comp Funct Genom* **3:** (5) 405.

Gavin AJ, Scheetz TE, Roberts CA, O'Leary B, Braun TA, Sheffield VC, Soares MB, Robinson JP, Casavant TL. 2002. Pooled library tissue tags for EST-based gene discovery. *Bioinformatics* **18:** (9) 1162.

George RA, Heringa J. 2002. Protein domain identification and improved sequence similarity searching using PSI-BLAST. *Protein Struct Funct Genet* **48:** (4) 672.

Goesmann A, Haubrock M, Meyer F, Kalinowski J, Giegerich R. 2002. PathFinder: Reconstruction and dynamic visualization of metabolic pathways. *Bioinformatics* **18:** (1) 124.

Gonnet P, Lisacek F. 2002. Probabilistic alignment of motifs with sequences. *Bioinformatics* **18:** (8) 1091.

Griffiths-Jones S, Bateman A. 2002. The use of structure information to increase alignment accuracy does not aid homologue detection with profile HMMs. *Bioinformatics* **18:** (9) 1243.

Haubold B, Wiehe T. 2002. Calculating the SNP-effective sample size from an alignment. *Bioinformatics* **18:** (1) 36.

Heringa J. 2002. Local weighting schemes for protein multiple sequence alignment. *Comput Chem* **26:** (5) 459.

Hollich V, Storm CEV, Sonnhammer ELL. 2002. OrthoGUI: graphical presentation of Orthostrapper results. *Bioinformatics* **18:** (9) 1272.

Hornquist M, Hertz J, Wahde M. 2002. Effective dimensionality of large-scale expression data using principal component analysis. *Biosystems* **65:** (2-3) 147.

Hwang DH, Schmitt WA, Stephanopoulos G, Stephanopoulos G. 2002. Determination of minimum sample size and discriminatory expression patterns in microarray data. *Bioinformatics* **18:** (9) 1184.

Issac B, Singh H, Kaur H, Raghava GPS. 2002. Locating probable genes using Fourier transform approach. *Bioinformatics* **18:** (1) 196.

Karchin R, Karplus K, Haussler D. 2002. Classifying G-protein coupled receptors with support vector machines. *Bioinformatics* **18:** (1) 147.

Kleinjung J, Douglas N, Heringa J. 2002. Parallelized multiple alignment. *Bioinformatics* **18:** (9) 1270.

Kochiwa H, Suzuki R, Washio T, Saito R, Bono H, Carninci P, Okazaki Y, Miki R, Hayashizaki Y, Tomita M. 2002. Inferring alternative splicing patterns in mouse from a full-length cDNA library and microarray data. *Genome Res* **12:** (8) 1286.

Kolesov G, Mewes HW, Frishman D. 2002. SNAPper: Gene order pre-

Copyright © 2003 John Wiley & Sons, Ltd. 

dicts gene function. *Bioinformatics* **18:** (7) 1017.
Kooperberg C, Sipione S, Le Blanc M, Strand AD, Cattaneo E, Olson JM. 2002. Microarray report special series. Evaluating test statistics to select interesting genes in microarray experiments. *Hum Mol Genet* **11:** (19) 2223.
Lambert C, Leonard N, De Bolle X, Depiereux E. 2002. ESyPred3D: Prediction of proteins 3D structures. *Bioinformatics* **18:** (9) 1250.
Li WT, Bernaola-Galvan P, Haghighi F, Grosse I. 2002. Applications of recursive segmentation to the analysis of DNA sequences. *Comput Chem* **26:** (5) 491.
Li WZ, Jaroszewski L, Godzik A. 2002. Tolerating some redundancy significantly speeds up clustering of large protein databases. *Bioinformatics* **18:** (1) 77.
Liebermeister W. 2002. Linear modes of gene expression determined by independent component analysis. *Bioinformatics* **18:** (1) 51.
Lillo F, Basile S, Mantegna RN. 2002. Comparative genomics study of inverted repeats in bacteria. *Bioinformatics* **18:** (7) 971.
Liu JF, Rost B. 2002. Target space for structural genomics revisited. *Bioinformatics* **18:** (7) 922.
Love B, Rank DR, Penn SG, Thomas RS. 2002. A conditional density error model for the statistical analysis of microarray data. *Bioinformatics* **18:** (8) 1064.
Macas J, Meszaros T, Nouzova M. 2002. PlantSat: A specialized database for plant satellite repeats. *Bioinformatics* **18:** (1) 28.
Medvedovic M, Sivaganesan S. 2002. Bayesian infinite mixture model based clustering of gene expression profiles. *Bioinformatics* **18:** (9) 1194.
Mendez MA, Hodar C, Vulpe C, Gonzalez M, Cambiazo V. 2002. Discriminant analysis to evaluate clustering of gene expression data. *FEBS Lett* **522:** (1-3) 24.
Moseyko N, Feldman LJ. 2002. VIZARD: Analysis of Affymetric *Arabidopsis* GeneChip® data. *Bioinformatics* **18:** (9) 1264.
Nguyen DV, Rocke DM. 2002. Tumor classification by partial least squares using microarray gene expression data. *Bioinformatics* **18:** (1) 39.
Nguyen DV, Rocke DM. 2002. Multi-class cancer classification *via* partial least squares with gene expression profiles. *Bioinformatics* **18:** (9) 1216.
Nishigaki K, Saito A. 2002. Genome structures embossed by oligonucleotide-stickiness. *Bioinformatics* **18:** (9) 1153.
Olshen AB, Jain AN. 2002. Deriving quantitative conclusions from microarray expression data. *Bioinformatics* **18:** (7) 961.
Osato N, Itoh M, Konno H, Kondo S, Shibata K, Carninci P, Shiraki T, Shinagawa A, Arakawa T, Kikuchi S, Sato K, Kawai J, Hayashizaki Y. 2002. A computer-based method of selecting clones for a full-length cDNA project: Simultaneous collection of negligibly redundant and variant cDNAs. *Genome Res* **12:** (7) 1127.
Ostermeier GC, Dix DJ, Krawetz SA. 2002. A bioinformatic strategy to rapidly characterize cDNA libraries. *Bioinformatics* **18:** (7) 949.
Park Y, Spouge JL. 2002. The correlation error and finite-size correction in an ungapped sequence alignment. *Bioinformatics* **18:** (9) 1236.
Pavlovic V, Garg A, Kasif S. 2002. A Bayesian framework for combining gene predictions. *Bioinformatics* **18:** (1) 19.
Peitsch MC. 2002. About the use of protein models. *Bioinformatics* **18:** (7) 934.
Perez AJ, Rodriguez A, Trelles O, Thode G. 2002. A computational strategy for protein function assignment which addresses the multidomain problem. *Comp Funct Genom* **3:** (5) 423.
Portugaly E, Kifer I, Linial M. 2002. Selecting targets for structural determination by navigating in a graph of protein families. *Bioinformatics* **18:** (7) 899.
Pupko T, Peer I, Hasegawa M, Graur D, Friedman N. 2002. A branch-and-bound algorithm for the inference of ancestral amino-acid sequences when the replacement rate varies among sites: Application to the evolution of five gene families. *Bioinformatics* **18:** (8) 1116.
Quentin Y, Chabalier J, Fichant G. 2002. Strategies for the identification, the assembly and the classification of integrated biological systems in completely sequenced genomes. *Comput Chem* **26:** (5) 447.
Radmacher MD, McShane LM, Simon R. 2002. A paradigm for class prediction using gene expression profiles. *J Comput Biol* **9:** (3) 505.
Ramoni MF, Sebatiani P, Kohane IS. 2002. Cluster analysis of gene expression dynamics. *Proc Natl Acad Sci U S A* **99:** (14) 9121.
Rhodes DR, Miller JC, Haab BB, Furge KA. 2002. CIT: Identification of differentially expressed clusters of genes from microarray data. *Bioinformatics* **18:** (1) 205.
Rifkin SA, Kim J. 2002. Geometry of gene expression dynamics. *Bioinformatics* **18:** (9) 1176.
Rogic S, Ouellette BFF, Mackworth AK. 2002. Improving gene recognition accuracy by combining predictions from two gene-finding programs. *Bioinformatics* **18:** (8) 1034.
Rohwer F, Edwards R. 2002. The Phage Proteomic Tree: A genome-based taxonomy for phage. *J Bacteriol* **184:** (16) 4529.
Rost U, Bornberg-Bauer E. 2002. TreeWiz: interactive exploration of huge trees. *Bioinformatics* **18:** (1) 109.
Rungsarityotin W, Khiripet N, Tanpraset C, Chitradon R. 2002. Grid computing and bioinformatics development. A case study on the *Oryza sativa* (rice) genome. *Pure Appl Chem* **74:** (6) 891.
Sakharkar MK, Kangueane P, Petrov DA, Kolaskar AS, Subbiah S. 2002. SEGE: A database on 'intron less/single exonic' genes from eukaryotes. *Bioinformatics* **18:** (9) 1266.
Samudrala R. 2002. Modeling genome structure and function. *Pure Appl Chem* **74:** (6) 907.
Sasik R, Iranfar N, Hwa T, Loomis WF. 2002. Extracting transcriptional events from temporal gene expression patterns during *Dictyostelium* development. *Bioinformatics* **18:** (1) 61.
Seo TK, Thorne JL, Hasegawa M, Kishino H. 2002. A viral sampling design for testing the molecular clock and for estimating evolutionary rates and divergence times. *Bioinformatics* **18:** (1) 115.
Shah AA, Giddings MC, Gesteland RF, Atkins JF, Ivanov IP. 2002. Computational identification of putative programmed translational frameshift sites. *Bioinformatics* **18:** (8) 1046.
Stephanopoulos G, Hwang DH, Schmitt WA, Misra J, Stephanopoulos G. 2002. Mapping physiological states from microarray expression measurements. *Bioinformatics* **18:** (8) 1054.
Storm CEV, Sonnhammer ELL. 2002. Automated ortholog inference from phylogenetic trees and calculation of orthology reliability. *Bioinformatics* **18:** (1) 92.
Strand AD, Olson JM, Kooperberg C. 2002. Microarray report special series. Estimating the statistical significance of gene expression changes observed with oligonucleotide arrays. *Hum Mol Genet* **11:** (19) 2207.
Stuart AC, Ilyin VA, Sali A. 2002. LigBase: A database of families of aligned ligand binding sites in known protein sequences and structures. *Bioinformatics* **18:** (1) 200.
Stuart GW, Moffett K, Baker S. 2002. Integrated gene and species phylogenies from unaligned whole genome protein sequences. *Bioinformatics* **18:** (1) 100.
Sturn A, Quackenbush J, Trajanoski Z. 2002. Genesis: Cluster analysis of microarray data. *Bioinformatics* **18:** (1) 207.
Tamames J, Clark D, Herrero J, Dopazo J, Blaschke C, Fernandez JM, Oliveros JC, Valencia A. 2002. Bioinformatics methods for the analysis of expression arrays: Data clustering and information extraction. *J Biotechnol* **98:** (2-3) 269.
Tanabe L, Wilbur WJ. 2002. Tagging gene and protein names in biomedical text. *Bioinformatics* **18:** (8) 1124.
Tomida S, Hanai T, Honda H, Kobayashi T. 2002. Analysis of expression profile using fuzzy adaptive resonance theory. *Bioinformatics* **18:** (8) 1073.
Tuffery P. 2002. CS-PSeq-Gen: Simulating the evolution of protein sequence under constraints. *Bioinformatics* **18:** (7) 1015.
Wang JB, Nygaard V, Smith-Sorensen B, Hovig E, Myklebost O. 2002. MArray: Analysing single, replicated or reversed microarray experiments. *Bioinformatics* **18:** (8) 1139.
Wang LC, Riethoven JJ, Robinson A. 2002. XEMBL: Distributing EMBL data in XML format. *Bioinformatics* **18:** (8) 1147.
Wolkenhauer O. 2002. Mathematical modelling in the post-genome era: Understanding genome expression and regulation: A system theoretic approach. *Biosystems* **65:** (1) 1.
Xuan ZY, McCombie WR, Zhang MQ. 2002. GFScan: A gene family search tool at genomic DNA level. *Genome Res* **12:** (7) 1142.
Yeramian E, Bonnefoy S, Langsley G. 2002. Physics-based gene identification: Proof of concept for *Plasmodium falciparum*. *Bioinformatics* **18:** (1) 190.
Zdobnov EM, Lopez R, Apweiler R, Etzold T. 2002. The EBI SRS server: New features. *Bioinformatics* **18:** (8) 1149.
Zhou Y, Huang GM, Wei LP. 2002. UniBLAST: A system to filter, cluster, and display BLAST results and assign unique gene annotation. *Bioinformatics* **18:** (9) 1268.

Copyright © 2003 John Wiley & Sons, Ltd. 

## References

[PDF_00874] Abascal Federico, Valencia Alfonso (2002). Clustering of proximal sequence space for the identification of protein families.. Bioinformatics.

[PDF_00776] Abdi Fadi A., Mundt Mark, Doggett Norman, Bradbury E. Morton, Chen Xian (2002). Validation of DNA sequences using mass spectrometry coupled with nucleoside mass tagging.. Genome Res.

[PDF_00779] Acestor Nathalie, Masina Slavica, Walker John, Saravia Nancy G., Fasel Nicolas, Quadroni Manfredo (2002). Establishing two-dimensional gels for the analysis of Leishmania proteomes.. Proteomics.

[PDF_00597] Adam Gregory C., Sorensen Erik J., Cravatt Benjamin F. (2002). Proteomic profiling of mechanistically distinct enzyme classes using a common chemotype.. Nat Biotechnol.

[PDF_00600] Agrawal Ganesh Kumar, Rakwal Randeep, Yonekura Masami, Kubo Akihiro, Saji Hikaru (2002). Proteome analysis of differentially displayed proteins as a tool for investigating ozone stress in rice (Oryza sativa L.) seedlings.. Proteomics.

[PDF_00438] Ahn Jung Hoon, Lee Yoon, Jeon ChoonJu, Lee Sang-Jin, Lee Byung-Hak, Choi Kang Duk, Bae Yong-Soo (2002). Identification of the genes differentially expressed in human dendritic cell subsets by cDNA subtraction and microarray analysis.. Blood.

[PDF_00218] Alevizos Ilias, Blaeser Bart, Gallagher George, Ohyama Hiroe, Wong David T. W., Todd Randy (2002). Odontogenic carcinoma: a functional genomic comparison with oral mucosal squamous cell carcinoma.. Oral Oncol.

[PDF_00445] Anisimov Sergey V., Tarasov Kirill V., Stern Michael D., Lakatta Edward G., Boheler Kenneth R. (2002). A quantitative and validated SAGE transcriptome reference for adult mouse heart.. Genomics.

[PDF_00087] Aparicio Samuel, Chapman Jarrod, Stupka Elia, Putnam Nik, Chia Jer-Ming, Dehal Paramvir, Christoffels Alan, Rash Sam, Hoon Shawn, Smit Arian (2002). Whole-genome shotgun assembly and analysis of the genome of Fugu rubripes.. Science.

[PDF_00007] Armstrong Scott A., Hsieh James J-D, Korsmeyer Stanley J. (2002). Genomic approaches to the pathogenesis and treatment of acute lymphoblastic leukemias.. Curr Opin Hematol.

[PDF_00603] Asirvatham Victor S., Watson Bonnie S., Sumner Lloyd W. (2002). Analytical and biological variances associated with proteomic studies of Medicago truncatula by two-dimensional polyacrylamide gel electrophoresis.. Proteomics.

[PDF_00448] Auger Sandrine, Danchin Antoine, Martin-Verstraete Isabelle (2002). Global expression profile of Bacillus subtilis grown in the presence of sulfate or methionine.. J Bacteriol.

[PDF_00385] Bagnall G., Plant M. A. (1991). HIV/AIDS risks, alcohol and illicit drug use among young adults in areas of high and low rates of HIV infection.. AIDS Care.

[PDF_00374] Bailey Jeannie, Manoil Colin (2002). Genome-wide internal tagging of bacterial exported proteins.. Nat Biotechnol.

[PDF_00878] Baisnée Pierre-François, Hampson Steve, Baldi Pierre (2002). Why are complementary DNA strands symmetric?. Bioinformatics.

[PDF_00397] Band Mark R., Olmstead Colleen, Everts Robin E., Liu Zonglin L., Lewin Harris A. (2002). A 3800 gene microarray for cattle functional genomics: comparison of gene expression in spleen, placenta, and brain.. Anim Biotechnol.

[PDF_00140] Bao Zhirong, Eddy Sean R. (2002). Automated de novo identification of repeat sequence families in sequenced genomes.. Genome Res.

[PDF_00719] Bardel Juliette, Louwagie Mathilde, Jaquinod Michel, Jourdain Agnès, Luche Sylvie, Rabilloud Thierry, Macherel David, Garin Jérôme, Bourguignon Jacques (2002). A survey of the plant mitochondrial proteome in relation to development.. Proteomics.

[PDF_00782] Bateman R. H., Carruthers R., Hoyes J. B., Jones C., Langridge J. I., Millar A., Vissers J. P. C. (2002). A novel precursor ion discovery method on a hybrid quadrupole orthogonal acceleration time-of-flight (Q-TOF) mass spectrometer for studying protein phosphorylation.. J Am Soc Mass Spectrom.

[PDF_00787] Battersby Bronwyn J., Lawrie Gwendolyn A., Johnston Angus P. R., Trau Matt (2002). Optical barcoding of colloidal suspensions: applications in genomics, proteomics and drug discovery.. Chem Commun (Camb).

[PDF_00451] Beliaev Alex S., Thompson Dorothea K., Fields Matthew W., Wu Liyou, Lies Douglas P., Nealson Kenneth H., Zhou Jizhong (2002). Microarray transcription profiling of a Shewanella oneidensis etrA mutant.. J Bacteriol.

[PDF_00010] Beroza Paul, Villar Hugo O., Wick Michael M., Martin Graeme R. (2002). Chemoproteomics as a basis for post-genomic drug discovery.. Drug Discov Today.

[PDF_00013] Betts Joanna C. (2002). Transcriptomics and proteomics: tools for the identification of novel drug targets and vaccine candidates for tuberculosis.. IUBMB Life.

[PDF_00607] Beyer Kirsten, Bardina Ludmilla, Grishina Galina, Sampson Hugh A. (2002). Identification of sesame seed allergens by 2-dimensional proteomics and Edman sequencing: seed storage proteins as common food allergens.. J Allergy Clin Immunol.

[PDF_00790] Bienvenut Willy V., Déon Catherine, Pasquarello Carla, Campbell Jennifer M., Sanchez Jean-Charles, Vestal Marvin L., Hochstrasser Denis F. (2002). Matrix-assisted laser desorption/ionization-tandem mass spectrometry with high resolution and sensitivity for identification and characterization of proteins.. Proteomics.

[PDF_00883] Bober Michael, Wiehe Kevin, Yung Christina, Onal Suzek Tugba, Lin Matthew, Baumgartner William, Winslow Raimond (2002). CaGE: cardiac gene expression knowledgebase.. Bioinformatics.

[PDF_00454] Bodyak Natalya, Kang Peter M., Hiromura Makoto, Sulijoadikusumo Indra, Horikoshi Nobuo, Khrapko Konstantin, Usheva Anny (2002). Gene expression profiling of the aging mouse cardiac myocytes.. Nucleic Acids Res.

[PDF_00886] Bonfield James K., Beal Kathryn F., Betts Matthew J., Staden Rodger (2002). Trev: a DNA trace editor and viewer.. Bioinformatics.

[PDF_00795] Bose Ajay K., Ing Yao Hain, Lavlinskaia Nina, Sareen Chaitanya, Pramanik Birendra N., Bartner Peter L., Liu Yan-Hui, Heimark Larry (2002). Microwave enhanced Akabori reaction for peptide analysis.. J Am Soc Mass Spectrom.

[PDF_00016] Botcherby Marc (2002). Harvesting the mouse genome.. Comp Funct Genomics.

[PDF_00723] Brambrink T., Wabnitz P., Halter R., Klocke R., Carnwath J., Kues W., Wrenzycki C., Paul D., Niemann H. (2002). Application of cDNA arrays to monitor mRNA profiles in single preimplantation mouse embryos.. Biotechniques.

[PDF_00018] Brizuela Leonardo, Richardson Aaron, Marsischky Gerald, Labaer Joshua (2002). The FLEXGene repository: exploiting the fruits of the genome projects by creating a needed resource to face the challenges of the post-genomic era.. Arch Med Res.

[PDF_00893] Bronner G., Spataro B., Page M., Gautier C., Rechenmann F. (2002). Modeling comparative mapping using objects and associations.. Comput Chem.

[PDF_00798] Brookes Paul S., Pinner Anita, Ramachandran Anup, Coward Lori, Barnes Stephen, Kim Helen, Darley-Usmar Victor M. (2002). High throughput two-dimensional blue-native electrophoresis: a tool for functional proteomics of mitochondria and signaling complexes.. Proteomics.

[PDF_00890] Broët Philippe, Richardson Sylvia, Radvanyi François (2002). Bayesian hierarchical model for identifying changes in gene expression from microarray experiments.. J Comput Biol.

[PDF_00091] Brugmans Bart, Fernandez del Carmen Asun, Bachem Christian W. B., van Os Hans, van Eck Herman J., Visser Richard G. F. (2002). A novel method for the construction of genome wide transcriptome maps.. Plant J.

[PDF_00022] Burbaum Jonathan, Tobal Gabriela M. (2002). Proteomics in drug discovery.. Curr Opin Chem Biol.

[PDF_00896] Buzko Oleksandr, Shokat Kevan M. (2002). A kinase sequence database: sequence alignments and family assignment.. Bioinformatics.

[PDF_00880] Bø Trond H., Jonassen Inge, Eidhammer Ingvar, Helgesen Carsten (2002). A fast top-down method for constructing reliable radiation hybrid frameworks.. Bioinformatics.

[PDF_00898] Cannata Nicola, Toppo Stefano, Romualdi Chiara, Valle Giorgio (2002). Simplifying amino acid alphabets by means of a branch and bound algorithm and substitution matrices.. Bioinformatics.

[PDF_00457] Cao Zhiyi, Wu Helen K., Bruce Amenda, Wollenberg Kurt, Panjwani Noorjahan (2002). Detection of differentially expressed genes in healing mouse corneas, using cDNA microarrays.. Invest Ophthalmol Vis Sci.

[PDF_00167] Carlton Jane M., Angiuoli Samuel V., Suh Bernard B., Kooij Taco W., Pertea Mihaela, Silva Joana C., Ermolaeva Maria D., Allen Jonathan E., Selengut Jeremy D., Koo Hean L. (2002). Genome sequence and comparative analysis of the model rodent malaria parasite Plasmodium yoelii yoelii.. Nature.

[PDF_00221] Carroll Joseph M., McElwee Kevin J., E King Lloyd, Byrne Michael C., Sundberg John P. (2002). Gene array profiling and immunomodulation studies define a cell-mediated immune response underlying the pathogenesis of alopecia areata in a mouse model and humans.. J Invest Dermatol.

[PDF_00460] Carson Daniel D., Lagow Errin, Thathiah Amantha, Al-Shami Rania, Farach-Carson Mary C., Vernon Michael, Yuan Lingwen, Fritz Marc A., Lessey Bruce (2002). Changes in gene expression during the early to mid-luteal (receptive phase) transition in human endometrium detected by high-density microarray screening.. Mol Hum Reprod.

[PDF_00611] Cavaletto Maria, Giuffrida Maria Gabriella, Fortunato Donatella, Gardano Laura, Dellavalle Giuseppina, Napolitano Lorenzo, Giunta Carlo, Bertino Enrico, Fabris Claudio, Conti Amedeo (2002). A proteomic approach to evaluate the butyrophilin gene family expression in human milk fat globule membrane.. Proteomics.

[PDF_00901] Chang Bill C. H., Halgamuge Saman K. (2002). Protein motif extraction with neuro-fuzzy optimization.. Bioinformatics.

[PDF_00903] Chapman Scott, Schenk Peer, Kazan Kemal, Manners John (2002). Using biplots to interpret gene expression patterns in plants.. Bioinformatics.

[PDF_00228] Chen Guoan, Gharib Tarek G., Huang Chiang-Ching, Thomas Dafydd G., Shedden Kerby A., Taylor Jeremy M. G., Kardia Sharon L. R., Misek David E., Giordano Thomas J., Iannettoni Mark D. (2002). Proteomic analysis of lung adenocarcinoma: identification of a highly expressed set of proteins in tumors.. Clin Cancer Res.

[PDF_00094] Chen Leslie Y. Y., Lu Szu-Hsien, Shih Edward S. C., Hwang Ming-Jing (2002). Single nucleotide polymorphism mapping using genome-wide unique sequences.. Genome Res.

[PDF_00905] Chen Shuo, Lesnik Elena A., Hall Thomas A., Sampath Rangarajan, Griffey Richard H., Ecker Dave J., Blyn Lawrence B. (2002). A bioinformatics based approach to discover small RNA genes in the Escherichia coli genome.. Biosystems.

[PDF_00908] Chen Yidong, Kamat Vishnu, Dougherty Edward R., Bittner Michael L., Meltzer Paul S., Trent Jeffery M. (2002). Ratio statistics of gene expression levels and applications to microarray data analysis.. Bioinformatics.

[PDF_00911] Chetouani Farid, Glaser Philippe, Kunst Frank (2002). DiffTool: building, visualizing and querying protein clusters.. Bioinformatics.

[PDF_00191] Christophides George K., Zdobnov Evgeny, Barillas-Mury Carolina, Birney Ewan, Blandin Stephanie, Blass Claudia, Brey Paul T., Collins Frank H., Danielli Alberto, Dimopoulos George (2002). Immunity-related genes and gene families in Anopheles gambiae.. Science.

[PDF_00913] Clare Amanda, King Ross D. (2002). Machine learning of functional class from phenotype data.. Bioinformatics.

[PDF_00915] Clark Terry, Lee Sanggyu, Ridgway Scott L., Wang San Ming (2002). Computational Analysis of Gene Identification with SAGE.. J Comput Biol.

[PDF_00236] Claudio Jaime O., Masih-Khan Esther, Tang Hongchang, Gonçalves Jason, Voralia Michael, Li Zhi Hua, Nadeem Vincent, Cukerman Eva, Francisco-Pabalan Ofelia, Liew Choong Chin (2002). A molecular compendium of genes expressed in multiple myeloma.. Blood.

[PDF_00920] Comet J. P., Henry J. (2002). Pairwise sequence alignment using a PROSITE pattern-derived similarity score.. Comput Chem.

[PDF_00922] Conant Gavin C., Wagner Andreas (2002). GenomeHistory: a software tool and its application to fully sequenced genomes.. Nucleic Acids Res.

[PDF_00925] Conklin Darrell, Jonassen Inge, Aasland Rein, Taylor William R. (2002). Association of nucleotide patterns with gene function classes: application to human 3' untranslated sequences.. Bioinformatics.

[PDF_00928] Contreras-Moreira B., Bates P. A. (2002). Domain fishing: a first step in protein comparative modelling.. Bioinformatics.

[PDF_00240] Cowen Leah E., Nantel André, Whiteway Malcolm S., Thomas David Y., Tessier Daniel C., Kohn Linda M., Anderson James B. (2002). Population genomics of drug resistance in Candida albicans.. Proc Natl Acad Sci U S A.

[PDF_00097] Cowman Alan F., Crabb Brendan S. (2002). The Plasmodium falciparum genome--a blueprint for erythrocyte invasion.. Science.

[PDF_00802] Craven Rachel A., Jackson David H., Selby Peter J., Banks Rosamonde E. (2002). Increased protein entry together with improved focussing using a combined IPGphor/Multiphor approach.. Proteomics.

[PDF_00615] Cronshaw Janet M., Krutchinsky Andrew N., Zhang Wenzhu, Chait Brian T., Matunis Michael J. (2002). Proteomic analysis of the mammalian nuclear pore complex.. J Cell Biol.

[PDF_00465] D'Agata Velia, Warren Stephen T., Zhao Weiqin, Torre Enrique R., Alkon Daniel L., Cavallaro Sebastiano (2002). Gene expression profiles in a transgenic animal model of fragile X syndrome.. Neurobiol Dis.

[PDF_00142] Daubin Vincent, Gouy Manolo, Perrière Guy (2002). A phylogenomic approach to bacterial phylogeny: evidence of a core of genes sharing a common history.. Genome Res.

[PDF_00933] De Groot Anne S., Sbai Hakima, Aubin Caitlin Saint, McMurry Julie, Martin William (2002). Immuno-informatics: Mining genomes for vaccine components.. Immunol Cell Biol.

[PDF_00936] Demir E., Babur O., Dogrusoz U., Gursoy A., Nisanci G., Cetin-Atalay R., Ozturk M. (2002). PATIKA: an integrated visual environment for collaborative construction and analysis of cellular pathways.. Bioinformatics.

[PDF_00145] Devos Katrien M., Brown James K. M., Bennetzen Jeffrey L. (2002). Genome size reduction through illegitimate recombination counteracts genome expansion in Arabidopsis.. Genome Res.

[PDF_00247] Diehn Maximilian, Alizadeh Ash A., Rando Oliver J., Liu Chih Long, Stankunas Kryn, Botstein David, Crabtree Gerald R., Brown Patrick O. (2002). Genomic expression programs and the integration of the CD28 costimulatory signal in T cell activation.. Proc Natl Acad Sci U S A.

[PDF_00940] Dodson J. M., Charles P. T., Stenger D. A., Pancrazio J. J. (2002). Quantitative assessment of filter-based cDNA microarrays: gene expression profiles of human T-lymphoma cell lines.. Bioinformatics.

[PDF_00727] Egger Boris, Leemans Ronny, Loop Thomas, Kammermeier Lars, Fan Yun, Radimerski Tanja, Strahm Martin C., Certa Ulrich, Reichert Heinrich (2002). Gliogenesis in Drosophila: genome-wide analysis of downstream genes of glial cells missing in the embryonic nervous system.. Development.

[PDF_00099] Eisen Jonathan A., Nelson Karen E., Paulsen Ian T., Heidelberg John F., Wu Martin, Dodson Robert J., Deboy Robert, Gwinn Michelle L., Nelson William C., Haft Daniel H. (2002). The complete genome sequence of Chlorobium tepidum TLS, a photosynthetic, anaerobic, green-sulfur bacterium.. Proc Natl Acad Sci U S A.

[PDF_00148] Eisen Jonathan A., Wu Martin (2002). Phylogenetic analysis and gene functional predictions: phylogenomics in action.. Theor Popul Biol.

[PDF_00618] El Fakhry Youssef, Ouellette Marc, Papadopoulou Barbara (2002). A proteomic approach to identify developmentally regulated proteins in Leishmania infantum.. Proteomics.

[PDF_00621] Eschenbrenner Michel, Wagner Mary Ann, Horn Troy A., Kraycer Jo Ann, Mujer Cesar V., Hagius Sue, Elzer Philip, DelVecchio Vito G. (2002). Comparative proteome analysis of Brucella melitensis vaccine strain Rev 1 and a virulent strain, 16M.. J Bacteriol.

[PDF_00472] Evans Simon J., Datson Nicole A., Kabbaj Mohamed, Thompson Robert C., Vreugdenhil Erno, De Kloet E. Ronald, Watson Stanley J., Akil Huda (2002). Evaluation of Affymetrix Gene Chip sensitivity in rat hippocampal tissue using SAGE analysis. Serial Analysis of Gene Expression.. Eur J Neurosci.

[PDF_00376] Fahrenkrug Scott C., Smith Timothy P. L., Freking Brad A., Cho Jennifer, White Joseph, Vallet Jeff, Wise Tommy, Rohrer Gary, Pertea Geo, Sultana Razvan (2002). Porcine gene discovery by normalized cDNA-library sequencing and EST cluster assembly.. Mamm Genome.

[PDF_00476] Fan Jinshui, Yang Xiaoling, Wang Wengong, Wood William H., Becker Kevin G., Gorospe Myriam (2002). Global analysis of stress-regulated mRNA turnover by using cDNA arrays.. Proc Natl Acad Sci U S A.

[PDF_00625] Fessler Michael B., Malcolm Kenneth C., Duncan Mark William, Worthen G. Scott (2002). A genomic and proteomic analysis of activation of the human neutrophil by lipopolysaccharide and its mediation by p38 mitogen-activated protein kinase.. J Biol Chem.

[PDF_00251] Fillmore G. Chris, Lin Zhaosheng, Bohling Sandra D., Robetorye Ryan S., Kim Chan-Hwan, Jenson Stephen D., Elenitoba-Johnson Kojo S. J., Lim Megan S. (2002). Gene expression profiling of cell lines derived from T-cell malignancies.. FEBS Lett.

[PDF_00629] Florens Laurence, Washburn Michael P., Raine J. Dale, Anthony Robert M., Grainger Munira, Haynes J. David, Moch J. Kathleen, Muster Nemone, Sacci John B., Tabb David L. (2002). A proteomic view of the Plasmodium falciparum life cycle.. Nature.

[PDF_00199] Forsyth Mark H., Cao Ping, Garcia Preston P., Hall Joshua D., Cover Timothy L. (2002). Genome-wide transcriptional profiling in a histidine kinase mutant of Helicobacter pylori identifies members of a regulon.. J Bacteriol.

[PDF_00479] Fowler Sarah, Thomashow Michael F. (2002). Arabidopsis transcriptome profiling indicates that multiple regulatory pathways are activated during cold acclimation in addition to the CBF cold response pathway.. Plant Cell.

[PDF_00943] Fradera Xavier, De La Cruz Xavier, Silva Carlos H. T. P., Gelpí Jose Luis, Luque F. J., Orozco Modesto (2002). Ligand-induced changes in the binding sites of proteins.. Bioinformatics.

[PDF_00946] Friedberg Iddo, Margalit Hanah (2002). PeCoP: automatic determination of persistently conserved positions in protein families.. Bioinformatics.

[PDF_00805] Fryksdale Beth G., Jedrzejewski Paul T., Wong David L., Gaertner Alfred L., Miller Brian S. (2002). Impact of deglycosylation methods on two-dimensional gel electrophoresis and matrix assisted laser desorption/ionization-time of flight-mass spectrometry for proteomic analysis.. Electrophoresis.

[PDF_00633] Fukao Youichiro, Hayashi Makoto, Nishimura Mikio (2002). Proteomic analysis of leaf peroxisomal proteins in greening cotyledons of Arabidopsis thaliana.. Plant Cell Physiol.

[PDF_00173] Fulton Theresa M., Van der Hoeven Rutger, Eannetta Nancy T., Tanksley Steven D. (2002). Identification, analysis, and utilization of conserved ortholog set markers for comparative genomics in higher plants.. Plant Cell.

[PDF_00706] Förster Jochen, Gombert Andreas Karoly, Nielsen Jens (2002). A functional genomics approach using metabolomics and in silico pathway analysis.. Biotechnol Bioeng.

[PDF_00949] Gadgil Chetan, Rink Anette, Beattie Craig, Hu Wei-Shou (2002). A mathematical model for suppression subtractive hybridization.. Comp Funct Genomics.

[PDF_00483] Garcia T., Roman-Roman S., Jackson A., Theilhaber J., Connolly T., Spinella-Jaegle S., Kawai S., Courtois B., Bushnell S., Auberval M. (2002). Behavior of osteoblast, adipocyte, and myoblast markers in genome-wide expression analysis of mouse calvaria primary osteoblasts in vitro.. Bone.

[PDF_00104] Gardner Malcolm J., Hall Neil, Fung Eula, White Owen, Berriman Matthew, Hyman Richard W., Carlton Jane M., Pain Arnab, Nelson Karen E., Bowman Sharen (2002). Genome sequence of the human malaria parasite Plasmodium falciparum.. Nature.

[PDF_00951] Gavin A. J., Scheetz T. E., Roberts C. A., O'Leary B., Braun T. A., Sheffield V. C., Soares M. B., Robinson J. P., Casavant T. L. (2002). Pooled library tissue tags for EST-based gene discovery.. Bioinformatics.

[PDF_00954] George Richard A., Heringa Jaap (2002). Protein domain identification and improved sequence similarity searching using PSI-BLAST.. Proteins.

[PDF_00254] Gharib Tarek G., Chen Guoan, Wang Hong, Huang Chiang-Ching, Prescott Michael S., Shedden Kerby, Misek David E., Thomas Dafydd G., Giordano Thomas J., Taylor Jeremy M. G. (2002). Proteomic analysis of cytokeratin isoforms uncovers association with survival in lung adenocarcinoma.. Neoplasia.

[PDF_00400] Giaever Guri, Chu Angela M., Ni Li, Connelly Carla, Riles Linda, Véronneau Steeve, Dow Sally, Lucau-Danila Ankuta, Anderson Keith, André Bruno (2002). Functional profiling of the Saccharomyces cerevisiae genome.. Nature.

[PDF_00957] Goesmann Alexander, Haubrock Martin, Meyer Folker, Kalinowski Jörn, Giegerich Robert (2002). PathFinder: reconstruction and dynamic visualization of metabolic pathways.. Bioinformatics.

[PDF_00960] Gonnet Pedro, Lisacek Frédérique (2002). Probabilistic alignment of motifs with sequences.. Bioinformatics.

[PDF_00258] Gordon Gavin J., Jensen Roderick V., Hsiao Li-Li, Gullans Steven R., Blumenstock Joshua E., Ramaswamy Sridhar, Richards William G., Sugarbaker David J., Bueno Raphael (2002). Translation of microarray data into clinically relevant cancer diagnostic tests using gene expression ratios in lung cancer and mesothelioma.. Cancer Res.

[PDF_00108] Gregory Simon G., Sekhon Mandeep, Schein Jacqueline, Zhao Shaying, Osoegawa Kazutoyo, Scott Carol E., Evans Richard S., Burridge Paul W., Cox Tony V., Fox Christopher A. (2002). A physical map of the mouse genome.. Nature.

[PDF_00962] Griffiths-Jones Sam, Bateman Alex (2002). The use of structure information to increase alignment accuracy does not aid homologue detection with profile HMMs.. Bioinformatics.

[PDF_00709] Grosu Paul, Townsend Jeffrey P., Hartl Daniel L., Cavalieri Duccio (2002). Pathway Processor: a tool for integrating whole-genome expression results into metabolic networks.. Genome Res.

[PDF_00024] Grünenfelder Björn, Winzeler Elizabeth A. (2002). Treasures and traps in genome-wide data sets: case examples from yeast.. Nat Rev Genet.

[PDF_00488] Guo Yueliang Leon, Chang Hsien-Chang, Tsai Jui-He, Huang Jong-Chi, Li Ching, Young Kung-Chia, Wu Li-Wha, Lai Ming-Der, Liu Hsiao-Sheng, Huang Wenya (2002). Two UVC-induced stress response pathways in HeLa cells identified by cDNA microarray.. Environ Mol Mutagen.

[PDF_00636] Gupta Surbhi, Pandit Shashi Bhushan, Srinivasan Narayanaswamy, Chatterji Dipankar (2002). Proteomics analysis of carbon-starved Mycobacterium smegmatis: induction of Dps-like protein.. Protein Eng.

[PDF_00269] Ha Geun Hyoung, Lee Seung Uook, Kang Deok Gyeong, Ha Na-Young, Kim Soon Hee, Kim Jina, Bae Jong Min, Kim Jae Won, Lee Chang-Won (2002). Proteome analysis of human stomach tissue: separation of soluble proteins by two-dimensional polyacrylamide gel electrophoresis and identification by mass spectrometry.. Electrophoresis.

[PDF_00202] Halfon Marc S., Grad Yonatan, Church George M., Michelson Alan M. (2002). Computation-based discovery of related transcriptional regulatory modules and motifs using an experimentally validated combinatorial model.. Genome Res.

[PDF_00274] Hartmann Karl Axel, Modlich Olga, Prisack Hans Bernd, Gerlach Bärbel, Bojar Hans (2002). Gene expression profiling of advanced head and neck squamous cell carcinomas and two squamous cell carcinoma cell lines under radio/chemotherapy using cDNA arrays.. Radiother Oncol.

[PDF_00278] Havlasová Jana, Hernychová Lenka, Halada Petr, Pellantová Vera, Krejsek Jan, Stulík Jirí, Macela Ales, Jungblut Peter R., Larsson Pär, Forsman Mats (2002). Mapping of immunoreactive antigens of Francisella tularensis live vaccine strain.. Proteomics.

[PDF_00492] Hayashi Mikiro, Mizoguchi Hiroshi, Shiraishi Norihiko, Obayashi Masaya, Nakagawa Satoshi, Imai Jun-ichi, Watanabe Shinya, Ota Toshio, Ikeda Masato (2002). Transcriptome analysis of acetate metabolism in Corynebacterium glutamicum using a newly developed metabolic array.. Biosci Biotechnol Biochem.

[PDF_00735] Heikinheimo K., Jee K. J., Niini T., Aalto Y., Happonen R-P, Leivo I., Knuutila S. (2002). Gene expression profiling of ameloblastoma and human tooth germ by means of a cDNA microarray.. J Dent Res.

[PDF_00027] Heller Michael J. (2002). DNA microarray technology: devices, systems, and applications.. Annu Rev Biomed Eng.

[PDF_00282] Hemby Scott E., Ginsberg Stephen D., Brunk Brian, Arnold Steven E., Trojanowski John Q., Eberwine James H. (2002). Gene expression profile for schizophrenia: discrete neuron transcription patterns in the entorhinal cortex.. Arch Gen Psychiatry.

[PDF_00810] Hering Joachim A., Innocent Peter R., Haris Parvez I. (2002). Automatic amide I frequency selection for rapid quantification of protein secondary structure from Fourier transform infrared spectra of proteins.. Proteomics.

[PDF_00967] Heringa Jaap (2002). Local weighting schemes for protein multiple sequence alignment.. Comput Chem.

[PDF_00031] Hochstrasser Denis F., Sanchez Jean-Charles, Appel Ron D. (2002). Proteomics and its trends facing nature's complexity.. Proteomics.

[PDF_00969] Hollich Volker, Storm Christian E. V., Sonnhammer Erik L. L. (2002). OrthoGUI: graphical presentation of Orthostrapper results.. Bioinformatics.

[PDF_00112] Holt Robert A., Subramanian G. Mani, Halpern Aaron, Sutton Granger G., Charlab Rosane, Nusskern Deborah R., Wincker Patrick, Clark Andrew G., Ribeiro José M. C., Wides Ron (2002). The genome sequence of the malaria mosquito Anopheles gambiae.. Science.

[PDF_00033] Holtorf Hauke, Guitton Marie-Christine, Reski Ralf (2002). Plant functional genomics.. Naturwissenschaften.

[PDF_00330] Howard G. C., Thomson C. S., Stroner P. L., Goodman C. M., Windsor P. M., Brewster D. H., Scottish Urological Oncology Group and Scottish Cancer Therapy Network (2001). Patterns of referral, management and survival of patients diagnosed with prostate cancer in Scotland during 1988 and 1993: results of a national, retrospective population-based audit.. BJU Int.

[PDF_00813] Huang Ying, Joo Sunghae, Duhon Melanie, Heller Michael, Wallace Bruce, Xu Xiao (2002). Dielectrophoretic cell separation and gene expression profiling on microelectronic chip arrays.. Anal Chem.

[PDF_00974] Hwang Daehee, Schmitt William A., Stephanopoulos George, Stephanopoulos Gregory (2002). Determination of minimum sample size and discriminatory expression patterns in microarray data.. Bioinformatics.

[PDF_00971] Hörnquist Michael, Hertz John, Wahde Mattias (2002). Effective dimensionality of large-scale expression data using principal component analysis.. Biosystems.

[PDF_00176] Iandolo John J., Worrell Veronica, Groicher Kajetan H., Qian Yudong, Tian Runying, Kenton Steve, Dorman Angela, Ji Honggui, Lin Shaoping, Loh Phoebe (2002). Comparative analysis of the genomes of the temperate bacteriophages phi 11, phi 12 and phi 13 of Staphylococcus aureus 8325.. Gene.

[PDF_00290] Imam-Sghiouar Naïma, Laude-Lemaire Isabelle, Labas Valérie, Pflieger Delphine, Le Caër Jean-Pierre, Caron Michel, Nabias Danièle Kerbiriou, Joubert-Caron Raymonde (2002). Subproteomics analysis of phosphorylated proteins: application to the study of B-lymphoblasts from a patient with Scott syndrome.. Proteomics.

[PDF_00380] Inaba Kazuo, Padma Potturi, Satouh Yuhkoh, Shin-I Tadasu, Kohara Yuji, Satoh Nori, Satou Yutaka (2002). EST analysis of gene expression in testis of the ascidian Ciona intestinalis.. Mol Reprod Dev.

[PDF_00977] Issac Biju, Singh Harpreet, Kaur Harpreet, Raghava G. P. S. (2002). Locating probable genes using Fourier transform approach.. Bioinformatics.

[PDF_00150] Jain Ravi, Rivera Maria C., Moore Jonathan E., Lake James A. (2002). Horizontal gene transfer in microbial genome evolution.. Theor Popul Biol.

[PDF_00294] Jazaeri Amir A., Yee Cindy J., Sotiriou Christos, Brantley Kelly R., Boyd Jeff, Liu Edison T. (2002). Gene expression profiles of BRCA1-linked, BRCA2-linked, and sporadic ovarian cancers.. J Natl Cancer Inst.

[PDF_00639] Jenkins L. W., Peters G. W., Dixon C. E., Zhang X., Clark R. S. B., Skinner J. C., Marion D. W., Adelson P. D., Kochanek P. M. (2002). Conventional and functional proteomics using large format two-dimensional gel electrophoresis 24 hours after controlled cortical impact in postnatal day 17 rats.. J Neurotrauma.

[PDF_00816] Jenssen Tor-Kristian, Langaas Mette, Kuo Winston P., Smith-Sørensen Birgitte, Myklebost Ola, Hovig Eivind (2002). Analysis of repeatability in spotted cDNA microarrays.. Nucleic Acids Res.

[PDF_00297] Jessani Nadim, Liu Yongsheng, Humphrey Mark, Cravatt Benjamin F. (2002). Enzyme activity profiles of the secreted and membrane proteome that depict cancer cell invasiveness.. Proc Natl Acad Sci U S A.

[PDF_00300] Ji Jiafu, Chen Xin, Leung Suet Yi, Chi Jen-Tsan A., Chu Kent Man, Yuen Siu Tsan, Li Rui, Chan Annie S. Y., Li Jiyou, Dunphy Nina (2002). Comprehensive analysis of the gene expression profiles in human gastric cancer cell lines.. Oncogene.

[PDF_00180] Jiang Zhihua, Melville Jenna S., Cao Honghe, Kumar Sudhir, Filipski Alan, Gibbins Ann M. Verrinder (2002). Measuring conservation of contiguous sets of autosomal markers on bovine and porcine genomes in relation to the map of the human genome.. Genome.

[PDF_00035] Jordan I. King, Rogozin Igor B., Wolf Yuri I., Koonin Eugene V. (2002). Microevolutionary genomics of bacteria.. Theor Popul Biol.

[PDF_00644] Journet Agnès, Chapel Agnès, Kieffer Sylvie, Roux Florence, Garin Jérôme (2002). Proteomic analysis of human lysosomes: application to monocytic and breast cancer cells.. Proteomics.

[PDF_00304] Juan Hsueh-Fen, Lin John Yi-Chung, Chang Wen-Hwei, Wu Chi-Yue, Pan Tai-Long, Tseng Min-Jen, Khoo Kay-Hooi, Chen Shui-Tein (2002). Biomic study of human myeloid leukemia cells differentiation to macrophages using DNA array, proteomic, and bioinformatic analytical methods.. Electrophoresis.

[PDF_00979] Karchin Rachel, Karplus Kevin, Haussler David (2002). Classifying G-protein coupled receptors with support vector machines.. Bioinformatics.

[PDF_00152] Karlin Samuel, Brocchieri Luciano, Trent Jonathan, Blaisdell B. Edwin, Mrázek Jan (2002). Heterogeneity of genome and proteome content in bacteria, archaea, and eukaryotes.. Theor Popul Biol.

[PDF_00308] Katsuma Susumu, Shiojima Satoshi, Hirasawa Akira, Takagaki Kazuchika, Kaminishi Yoshinori, Koba Masahiro, Hagidai Yoshimi, Murai Masatoshi, Ohgi Tadaaki, Yano Junichi (2002). Global analysis of differentially expressed genes during progression of calcium oxalate nephrolithiasis.. Biochem Biophys Res Commun.

[PDF_00040] Kennedy Sandy (2002). The role of proteomics in toxicology: identification of biomarkers of toxicity by protein expression analysis.. Biomarkers.

[PDF_00496] Kita Hiroko, Carmichael Jenny, Swartz Jina, Muro Shizuko, Wyttenbach Andreas, Matsubara Kenichi, Rubinsztein David C., Kato Kikuya (2002). Modulation of polyglutamine-induced cell death by genes identified by expression profiling.. Hum Mol Genet.

[PDF_00500] Kitagawa Emiko, Takahashi Junko, Momose Yuko, Iwahashi Hitoshi (2002). Effects of the pesticide thiuram: genome-wide screening of indicator genes by yeast DNA microarray.. Environ Sci Technol.

[PDF_00313] Kitahara Osamu, Katagiri Toyomasa, Tsunoda Tatsuhiko, Harima Yoko, Nakamura Yusuke (2002). Classification of sensitivity or resistance of cervical cancers to ionizing radiation according to expression profiles of 62 genes selected by cDNA microarray analysis.. Neoplasia.

[PDF_00043] Kleerebezem Michiel, Boels Ingeborg C., Groot Masja Nierop, Mierau Igor, Sybesma Wilbert, Hugenholtz Jeroen (2002). Metabolic engineering of Lactococcus lactis: the impact of genomics and metabolic modelling.. J Biotechnol.

[PDF_00981] Kleinjung Jens, Douglas Nigel, Heringa Jaap (2002). Parallelized multiple alignment.. Bioinformatics.

[PDF_00983] Kochiwa Hiromi, Suzuki Ryosuke, Washio Takanori, Saito Rintaro, Bono Hidemasa, Carninci Piero, Okazaki Yasushi, Miki Rika, Hayashizaki Yoshihide, Tomita Masaru (2002). Inferring alternative splicing patterns in mouse from a full-length cDNA library and microarray data.. Genome Res.

[PDF_00647] Koller Antonius, Washburn Michael P., Lange B. Markus, Andon Nancy L., Deciu Cosmin, Haynes Paul A., Hays Lara, Schieltz David, Ulaszek Ryan, Wei Jing (2002). Proteomic survey of metabolic pathways in rice.. Proc Natl Acad Sci U S A.

[PDF_00991] Kooperberg Charles, Sipione Simonetta, LeBlanc Michael, Strand Andrew D., Cattaneo Elena, Olson James M. (2002). Evaluating test statistics to select interesting genes in microarray experiments.. Hum Mol Genet.

[PDF_00383] Koumproglou Rachil, Wilkes Tim M., Townson Paul, Wang Xiao Y., Beynon Jim, Pooni Harpal S., Newbury H. John, Kearsey Mike J. (2002). STAIRS: a new genetic resource for functional genomic studies of Arabidopsis.. Plant J.

[PDF_00503] Kubo Tateki, Yamashita Toshihide, Yamaguchi Atsushi, Hosokawa Ko, Tohyama Masaya (2002). Analysis of genes induced in peripheral nerve after axotomy using cDNA microarrays.. J Neurochem.

[PDF_00129] Kutsenko Alexey S., Gizatullin Rinat Z., Al-Amin Ali N., Wang Fuli, Kvasha Sergei M., Podowski Raf M., Matushkin Yuri G., Gyanchandani Anita, Muravenko Olga V., Levitsky Viktor G. (2002). NotI flanking sequences: a tool for gene discovery and verification of the human genome.. Nucleic Acids Res.

[PDF_00995] Lambert Christophe, Léonard Nadia, De Bolle Xavier, Depiereux Eric (2002). ESyPred3D: Prediction of proteins 3D structures.. Bioinformatics.

[PDF_00650] Lasonder Edwin, Ishihama Yasushi, Andersen Jens S., Vermunt Adriaan M. W., Pain Arnab, Sauerwein Robert W., Eling Wijnand M. C., Hall Neil, Waters Andrew P., Stunnenberg Hendrik G. (2002). Analysis of the Plasmodium falciparum proteome by high-accuracy mass spectrometry.. Nature.

[PDF_00819] Lee Hookeun, Griffin Timothy J., Gygi Steven P., Rist Beate, Aebersold Ruedi (2002). Development of a multiplexed microcapillary liquid chromatography system for high-throughput proteome analysis.. Anal Chem.

[PDF_00403] Lenaers Guy, Pelloquin Laetitia, Olichon Aurélien, Emorine Laurent J., Guillou Emmanuelle, Delettre Cécile, Hamel Christian P., Ducommun Bernard, Belenguer Pascale (2002). What similarity between human and fission yeast proteins is required for orthology?. Yeast.

[PDF_00822] Lesaicherre Marie-Laure, Lue Rina Y. P., Chen Grace Y. J., Zhu Qing, Yao Shao Q. (2002). Intein-mediated biotinylation of proteins and its application in a protein microarray.. J Am Chem Soc.

[PDF_00692] Lesley Scott A., Kuhn Peter, Godzik Adam, Deacon Ashley M., Mathews Irimpan, Kreusch Andreas, Spraggon Glen, Klock Heath E., McMullan Daniel, Shin Tanya (2002). Structural genomics of the Thermotoga maritima proteome implemented in a high-throughput structure determination pipeline.. Proc Natl Acad Sci U S A.

[PDF_00155] Lespinet Olivier, Wolf Yuri I., Koonin Eugene V., Aravind L. (2002). The role of lineage-specific gene family expansion in the evolution of eukaryotes.. Genome Res.

[PDF_00506] Levsky Jeffrey M., Shenoy Shailesh M., Pezo Rossanna C., Singer Robert H. (2002). Single-cell gene expression profiling.. Science.

[PDF_00320] Li Jinong, Zhang Zhen, Rosenzweig Jason, Wang Young Y., Chan Daniel W. (2002). Proteomics and bioinformatics approaches for identification of serum biomarkers to detect breast cancer.. Clin Chem.

[PDF_00997] Li Wentian, Bernaola-Galván Pedro, Haghighi Fatameh, Grosse Ivo (2002). Applications of recursive segmentation to the analysis of DNA sequences.. Comput Chem.

[PDF_00323] Li Yao, Li Yali, Tang Rong, Xu Hong, Qiu Minyan, Chen Qin, Chen Juxiang, Fu Zhiren, Ying Kang, Xie Yi (2002). Discovery and analysis of hepatocellular carcinoma genes using cDNA microarrays.. J Cancer Res Clin Oncol.

[PDF_01005] Lillo Fabrizio, Basile Salvatore, Mantegna Rosario N. (2002). Comparative genomics study of inverted repeats in bacteria.. Bioinformatics.

[PDF_00654] Lipton Mary S., Pasa-Tolic' Ljiljana, Anderson Gordon A., Anderson David J., Auberry Deanna L., Battista John R., Daly Michael J., Fredrickson Jim, Hixson Kim K., Kostandarithes Heather (2002). Global analysis of the Deinococcus radiodurans proteome by using accurate mass tags.. Proc Natl Acad Sci U S A.

[PDF_01007] Liu Jinfeng, Rost Burkhard (2002). Target space for structural genomics revisited.. Bioinformatics.

[PDF_00049] Lorenz Michael C. (2002). Genomic approaches to fungal pathogenicity.. Curr Opin Microbiol.

[PDF_01009] Love Brad, Rank David R., Penn Sharron G., Jenkins David A., Thomas Russell S. (2002). A conditional density error model for the statistical analysis of microarray data.. Bioinformatics.

[PDF_00327] Luo L-Y, Herrera I., Soosaipillai A., Diamandis E. P. (2002). Identification of heat shock protein 90 and other proteins as tumour antigens by serological screening of an ovarian carcinoma expression library.. Br J Cancer.

[PDF_00047] López-Otín Carlos, Overall Christopher M. (2002). Protease degradomics: a new challenge for proteomics.. Nat Rev Mol Cell Biol.

[PDF_00739] Ma Xianyong, Husain Tupur, Peng Hui, Lin Sharon, Mironenko Olga, Maun Noel, Johnson Steven, Tuck David, Berliner Nancy, Krause Diane S. (2002). Development of a murine hematopoietic progenitor complementary DNA microarray using a subtracted complementary DNA library.. Blood.

[PDF_00051] Macgregor Pascale F., Squire Jeremy A. (2002). Application of microarrays to the analysis of gene expression in cancer.. Clin Chem.

[PDF_00697] Mallick Parag, Boutz Daniel R., Eisenberg David, Yeates Todd O. (2002). Genomic evidence that the intracellular proteins of archaeal microbes contain disulfide bonds.. Proc Natl Acad Sci U S A.

[PDF_00828] Manduchi Elisabetta, Scearce L. Marie, Brestelli John E., Grant Gregory R., Kaestner Klaus H., Stoeckert Christian J. (2002). Comparison of different labeling methods for two-channel high-density microarray experiments.. Physiol Genomics.

[PDF_00514] Maurer Martin H., Frietsch Thomas, Waschke Klaus F., Kuschinsky Wolfgang, Gassmann Max, Schneider Armin (2002). Cerebral transcriptome analysis of transgenic mice overexpressing erythropoietin.. Neurosci Lett.

[PDF_00053] McCormack Grace P., Clewley Jonathan P. (2002). The application of molecular phylogenetics to the analysis of viral genome diversity and evolution.. Rev Med Virol.

[PDF_00331] McDonough Jason L., Neverova Irina, Van Eyk Jennifer E. (2002). Proteomic analysis of human biopsy samples by single two-dimensional electrophoresis: Coomassie, silver, mass spectrometry, and Western blotting.. Proteomics.

[PDF_01014] Medvedovic Mario, Sivaganesan Siva (2002). Bayesian infinite mixture model based clustering of gene expression profiles.. Bioinformatics.

[PDF_00517] Misra Jatin, Schmitt William, Hwang Daehee, Hsiao Li-Li, Gullans Steve, Stephanopoulos George, Stephanopoulos Gregory (2002). Interactive exploration of microarray gene expression patterns in a reduced dimensional space.. Genome Res.

[PDF_00658] Misumi Shogo, Fuchigami Takashi, Takamune Nobutoki, Takahashi Ichiro, Takama Michiho, Shoji Shozo (2002). Three isoforms of cyclophilin A associated with human immunodeficiency virus type 1 were found by proteomics by using two-dimensional gel electrophoresis and matrix-assisted laser desorption ionization-time of flight mass spectrometry.. J Virol.

[PDF_00663] Molloy Mark P., Phadke Nikhil D., Chen Hong, Tyldesley Richard, Garfin David E., Maddock Janine R., Andrews Philip C. (2002). Profiling the alkaline membrane proteome of Caulobacter crescentus with two-dimensional electrophoresis and mass spectrometry.. Proteomics.

[PDF_00743] Moran Jennifer L., Li Yizheng, Hill Andrew A., Mounts William M., Miller Christopher P. (2002). Gene expression changes during mouse skeletal myoblast differentiation revealed by transcriptional profiling.. Physiol Genomics.

[PDF_01020] Moseyko Nick, Feldman Lewis J. (2002). VIZARD: analysis of Affymetrix Arabidopsis GeneChip data.. Bioinformatics.

[PDF_00521] Muffler Andrea, Bettermann Sandra, Haushalter Michael, Hörlein Andreas, Neveling Ute, Schramm Maren, Sorgenfrei Oliver (2002). Genome-wide transcription profiling of Corynebacterium glutamicum after heat shock and during growth on acetate and glucose.. J Biotechnol.

[PDF_00511] Mäder Ulrike, Homuth Georg, Scharf Christian, Büttner Knut, Bode Rüdiger, Hecker Michael (2002). Transcriptome and proteome analysis of Bacillus subtilis gene expression modulated by amino acid availability.. J Bacteriol.

[PDF_01022] Nguyen Danh V., Rocke David M. (2002). Multi-class cancer classification via partial least squares with gene expression profiles.. Bioinformatics.

[PDF_01028] Nishigaki Koichi, Saito Ayumu (2002). Genome structures embossed by oligonucleotide-stickiness.. Bioinformatics.

[PDF_00825] Ogorzalek Loo Rachel R., Loo Joseph A., Du Ping, Holler Tod (2002). In vivo labeling: a glimpse of the dynamic proteome and additional constraints for protein identification.. J Am Soc Mass Spectrom.

[PDF_00525] Okinaka Yasushi, Yang Ching-Hong, Perna Nicole T., Keen Noel T. (2002). Microarray profiling of Erwinia chrysanthemi 3937 genes that are regulated during plant infection.. Mol Plant Microbe Interact.

[PDF_00528] Okuda Tomohiko, Sumiya Toshiki, Iwai Naoharu, Miyata Toshiyuki (2002). Difference of gene expression profiles in spontaneous hypertensive rats and Wistar-Kyoto rats from two sources.. Biochem Biophys Res Commun.

[PDF_01030] Olshen Adam B., Jain Ajay N. (2002). Deriving quantitative conclusions from microarray expression data.. Bioinformatics.

[PDF_01032] Osato Naoki, Itoh Masayoshi, Konno Hideaki, Kondo Shinji, Shibata Kazuhiro, Carninci Piero, Shiraki Toshiyuki, Shinagawa Akira, Arakawa Takahiro, Kikuchi Shoshi (2002). A computer-based method of selecting clones for a full-length cDNA project: simultaneous collection of negligibly redundant and variant cDNAs.. Genome Res.

[PDF_00531] Oshima Taku, Wada Chieko, Kawagoe Yuya, Ara Takeshi, Maeda Maki, Masuda Yasushi, Hiraga Sota, Mori Hirotada (2002). Genome-wide analysis of deoxyadenosine methyltransferase-mediated control of gene expression in Escherichia coli.. Mol Microbiol.

[PDF_01037] Ostermeier G. Charles, Dix David J., Krawetz Stephen A. (2002). A bioinformatic strategy to rapidly characterize cDNA libraries.. Bioinformatics.

[PDF_00056] Panek Heather, O'Brian Mark R. (2002). A whole genome view of prokaryotic haem biosynthesis.. Microbiology.

[PDF_00832] Panfilov Oleg, Lanne Boel (2002). Peptide mass fingerprinting from wet and dry two-dimensional gels and its application in proteomics.. Anal Biochem.

[PDF_01039] Park Yonil, Spouge John L. (2002). The correlation error and finite-size correction in an ungapped sequence alignment.. Bioinformatics.

[PDF_01043] Peitsch Manuel C. (2002). About the use of protein models.. Bioinformatics.

[PDF_00338] Piquemal David, Commes Thérèse, Manchon Laurent, Lejeune Mireille, Ferraz Conchita, Pugnère Denis, Demaille Jacques, Elalouf Jean-Marc, Marti Jacques (2002). Transcriptome analysis of monocytic leukemia cell differentiation.. Genomics.

[PDF_00712] Plumb Robert S., Stumpf Chris L., Gorenstein Marc V., Castro-Perez Jose M., Dear Gordon J., Anthony Maria, Sweatman Brian C., Connor Susan C., Haselden John N. (2002). Metabonomics: the use of electrospray mass spectrometry coupled to reversed-phase liquid chromatography shows potential for the screening of rat urine in drug development.. Rapid Commun Mass Spectrom.

[PDF_01048] Portugaly Elon, Kifer Ilona, Linial Michal (2002). Selecting targets for structural determination by navigating in a graph of protein families.. Bioinformatics.

[PDF_00343] Pucci-Minafra Ida, Fontana Simona, Cancemi Patrizia, Basiricò Loredana, Caricato Silvana, Minafra Salvatore (2002). A contribution to breast cancer cell proteomics: detection of new sequences.. Proteomics.

[PDF_01051] Pupko Tal, Pe'er Itsik, Hasegawa Masami, Graur Dan, Friedman Nir (2002). A branch-and-bound algorithm for the inference of ancestral amino-acid sequences when the replacement rate varies among sites: Application to the evolution of five gene families.. Bioinformatics.

[PDF_00702] Pédelacq Jean-Denis, Piltch Emily, Liong Elaine C., Berendzen Joel, Kim Chang-Yub, Rho Beom-Seop, Park Min S., Terwilliger Thomas C., Waldo Geoffrey S. (2002). Engineering soluble proteins for structural genomics.. Nat Biotechnol.

[PDF_01045] Pérez A. J., Rodríguez A., Trelles O., Thode G. (2002). A computational strategy for protein function assignment which addresses the multidomain problem.. Comp Funct Genomics.

[PDF_01055] Quentin Yves, Chabalier Julie, Fichant Gwennaele (2002). Strategies for the identification, the assembly and the classification of integrated biological systems in completely sequenced genomes.. Comput Chem.

[PDF_00058] Quinn-Senger Kerry E., Ramachandran Ravi, Rininger Joseph A., Kelly Karen M., Lewin David A. (2002). Staking out novelty on the genomic frontier.. Curr Opin Chem Biol.

[PDF_01058] Radmacher Michael D., McShane Lisa M., Simon Richard (2002). A paradigm for class prediction using gene expression profiles.. J Comput Biol.

[PDF_01060] Ramoni Marco F., Sebastiani Paola, Kohane Isaac S. (2002). Cluster analysis of gene expression dynamics.. Proc Natl Acad Sci U S A.

[PDF_00667] Rappsilber Juri, Ryder Ursula, Lamond Angus I., Mann Matthias (2002). Large-scale proteomic analysis of the human spliceosome.. Genome Res.

[PDF_00670] Rashid K. Aftab, Hevi Sarah, Chen Yin, Le Cahérec Francoise, Chuck Steven L. (2002). A proteomic approach identifies proteins in hepatocytes that bind nascent apolipoprotein B.. J Biol Chem.

[PDF_00206] Remaley Alan T., Bark Samantha, Walts Avram D., Freeman Lita, Shulenin Sergey, Annilo Tarmo, Elgin Eric, Rhodes Hope E., Joyce Charles, Dean Michael (2002). Comparative genome analysis of potential regulatory elements in the ABCG5-ABCG8 gene cluster.. Biochem Biophys Res Commun.

[PDF_00835] Renault Jean Philippe, Bernard André, Juncker David, Michel Bruno, Bosshard Hans Rudolf, Delamarche Emmanuel (2002). Fabricating microarrays of functional proteins using affinity contact printing.. Angew Chem Int Ed Engl.

[PDF_00746] Rho Jaerang, Altmann Curtis R., Socci Nicholas D., Merkov Lubomir, Kim Nacksung, So Hongseob, Lee Okbok, Takami Masamichi, Brivanlou Ali H., Choi Yongwon (2002). Gene expression profiling of osteoclast differentiation by combined suppression subtractive hybridization (SSH) and cDNA microarray analysis.. DNA Cell Biol.

[PDF_00346] Rhodes Daniel R., Barrette Terrence R., Rubin Mark A., Ghosh Debashis, Chinnaiyan Arul M. (2002). Meta-analysis of microarrays: interstudy validation of gene expression profiles reveals pathway dysregulation in prostate cancer.. Cancer Res.

[PDF_01062] Rhodes Daniel R., Miller Jeremy C., Haab Brian B., Furge Kyle A. (2002). CIT: identification of differentially expressed clusters of genes from microarray data.. Bioinformatics.

[PDF_01065] Rifkin S. A., Kim J. (2002). Geometry of gene expression dynamics.. Bioinformatics.

[PDF_00751] Rivolta Marcelo N., Halsall Antony, Johnson Claire M., Tones Michael A., Holley Matthew C. (2002). Transcript profiling of functionally related groups of genes during conditional differentiation of a mammalian cochlear hair cell line.. Genome Res.

[PDF_00210] Rodríguez-Navarro Susana, Llorente Bertrand, Rodríguez-Manzaneque María Teresa, Ramne Anna, Uber Genoveva, Marchesan Denis, Dujon Bernard, Herrero Enrique, Sunnerhagen Per, Pérez-Ortín José E. (2002). Functional analysis of yeast gene families involved in metabolism of vitamins B1 and B6.. Yeast.

[PDF_01067] Rogic Sanja, Ouellette B. F. Francis, Mackworth Alan K. (2002). Improving gene recognition accuracy by combining predictions from two gene-finding programs.. Bioinformatics.

[PDF_01070] Rohwer Forest, Edwards Rob (2002). The Phage Proteomic Tree: a genome-based taxonomy for phage.. J Bacteriol.

[PDF_00838] Ross Andrew R. S., Lee Peter J., Smith Duncan L., Langridge James I., Whetton Anthony D., Gaskell Simon J. (2002). Identification of proteins from two-dimensional polyacrylamide gels using a novel acid-labile surfactant.. Proteomics.

[PDF_01072] Rost Ursula, Bornberg-Bauer Erich (2002). TreeWiz: interactive exploration of huge trees.. Bioinformatics.

[PDF_00535] Roth Stephen M., Ferrell Robert E., Peters David G., Metter E. Jeffrey, Hurley Ben F., Rogers Marc A. (2002). Influence of age, sex, and strength training on human muscle gene expression determined by microarray.. Physiol Genomics.

[PDF_00755] Ruuska Sari A., Girke Thomas, Benning Christoph, Ohlrogge John B. (2002). Contrapuntal networks of gene expression during Arabidopsis seed filling.. Plant Cell.

[PDF_01077] Sakharkar Meena K., Kangueane Pandjassarame, Petrov Dmitri A., Kolaskar A. S., Subbiah S. (2002). SEGE: A database on 'intron less/single exonic' genes from eukaryotes.. Bioinformatics.

[PDF_00412] Scearce L. Marie, Brestelli John E., McWeeney Shannon K., Lee Catherine S., Mazzarelli Joan, Pinney Deborah F., Pizarro Angel, Stoeckert Christian J., Clifton Sandra W., Permutt M. Alan (2002). Functional genomics of the endocrine pancreas: the pancreas clone set and PancChip, new resources for diabetes research.. Diabetes.

[PDF_00843] Schlosser Andreas, Bodem Jochen, Bossemeyer Dirk, Grummt Ingrid, Lehmann Wolf Dieter (2002). Identification of protein phosphorylation sites by combination of elastase digestion, immobilized metal affinity chromatography, and quadrupole-time of flight tandem mass spectrometry.. Proteomics.

[PDF_00061] Schofield Deborah, Triche Timothy J. (2002). cDNA microarray analysis of global gene expression in sarcomas.. Curr Opin Oncol.

[PDF_00063] Schultze Joachim L., Bohlen Heribert (2002). Influence of genomics on cancer vaccine development from guess to prediction.. Curr Pharm Des.

[PDF_00350] Schwertz Hansjörg, Längin Tina, Platsch Herbert, Richert Joachim, Bomm Sabine, Schmidt Martin, Hillen Heinz, Blaschke Gottfried, Meyer Jürgen, Darius Harald (2002). Two-dimensional analysis of myocardial protein expression following myocardial ischemia and reperfusion in rabbits.. Proteomics.

[PDF_00539] Seki Motoaki, Narusaka Mari, Ishida Junko, Nanjo Tokihiko, Fujita Miki, Oono Youko, Kamiya Asako, Nakajima Maiko, Enju Akiko, Sakurai Tetsuya (2002). Monitoring the expression profiles of 7000 Arabidopsis genes under drought, cold and high-salinity stresses using a full-length cDNA microarray.. Plant J.

[PDF_01085] Seo Tae-Kun, Thorne Jeffrey L., Hasegawa Masami, Kishino Hirohisa (2002). A viral sampling design for testing the molecular clock and for estimating evolutionary rates and divergence times.. Bioinformatics.

[PDF_01088] Shah Atul A., Giddings Michael C., Parvaz Jasmin B., Gesteland Raymond F., Atkins John F., Ivanov Ivaylo P. (2002). Computational identification of putative programmed translational frameshift sites.. Bioinformatics.

[PDF_00851] Shen Yufeng, Zhao Rui, Berger Scott J., Anderson Gordon A., Rodriguez Nestor, Smith Richard D. (2002). High-efficiency nanoscale liquid chromatography coupled on-line with mass spectrometry using nanoelectrospray ionization for proteomics.. Anal Chem.

[PDF_00066] Shimamoto Ko, Kyozuka Junko (2002). Rice as a model for comparative genomics of plants.. Annu Rev Plant Biol.

[PDF_00758] Simin Karl, Scuderi Anne, Reamey James, Dunn Diane, Weiss Robert, Metherall James E., Letsou Anthea (2002). Profiling patterned transcripts in Drosophila embryos.. Genome Res.

[PDF_00543] Sipione Simonetta, Rigamonti Dorotea, Valenza Marta, Zuccato Chiara, Conti Luciano, Pritchard Joel, Kooperberg Charles, Olson James M., Cattaneo Elena (2002). Early transcriptional profiles in huntingtin-inducible striatal cells by microarray analyses.. Hum Mol Genet.

[PDF_00673] Skynner Heather A., Rosahl Thomas W., Knowles Michael R., Salim Kamran, Reid Lee, Cothliff Rosa, McAllister George, Guest Paul C. (2002). Alterations of stress related proteins in genetically altered mice revealed by two-dimensional differential in-gel electrophoresis analysis.. Proteomics.

[PDF_00551] Smith Jennifer J., Marelli Marcello, Christmas Rowan H., Vizeacoumar Franco J., Dilworth David J., Ideker Trey, Galitski Timothy, Dimitrov Krassen, Rachubinski Richard A., Aitchison John D. (2002). Transcriptome profiling to identify genes involved in peroxisome assembly and function.. J Cell Biol.

[PDF_00387] Sonstegard Tad S., Capuco Anthony V., White Joseph, Van Tassell Curtis P., Connor Erin E., Cho Jennifer, Sultana Razvan, Shade Larry, Wray James E., Wells Kevin D. (2002). Analysis of bovine mammary gland EST and functional annotation of the Bos taurus gene index.. Mamm Genome.

[PDF_00068] Srinivas Pothur R., Verma Mukesh, Zhao Yinming, Srivastava Sudhir (2002). Proteomics for cancer biomarker discovery.. Clin Chem.

[PDF_01091] Stephanopoulos Gregory, Hwang Daehee, Schmitt William A., Misra Jatin, Stephanopoulos George (2002). Mapping physiological states from microarray expression measurements.. Bioinformatics.

[PDF_01097] Strand Andrew D., Olson James M., Kooperberg Charles (2002). Estimating the statistical significance of gene expression changes observed with oligonucleotide arrays.. Hum Mol Genet.

[PDF_01101] Stuart Ashley C., Ilyin Valentin A., Sali Andrej (2002). LigBase: a database of families of aligned ligand binding sites in known protein sequences and structures.. Bioinformatics.

[PDF_01104] Stuart Gary W., Moffett Karen, Baker Steve (2002). Integrated gene and species phylogenies from unaligned whole genome protein sequences.. Bioinformatics.

[PDF_01107] Sturn Alexander, Quackenbush John, Trajanoski Zlatko (2002). Genesis: cluster analysis of microarray data.. Bioinformatics.

[PDF_00133] Swan Kathryn A., Curtis Damian E., McKusick Kathleen B., Voinov Alexander V., Mapa Felipa A., Cancilla Michael R. (2002). High-throughput gene mapping in Caenorhabditis elegans.. Genome Res.

[PDF_01109] Tamames Javier, Clark Dominic, Herrero Javier, Dopazo Joaquín, Blaschke Christian, Fernández José M., Oliveros Juan C., Valencia Alfonso (2002). Bioinformatics methods for the analysis of expression arrays: data clustering and information extraction.. J Biotechnol.

[PDF_00761] Tan Seong-Seng, Gunnersen Jenny, Job Christopher (2002). Global gene expression analysis of developing neocortex using SAGE.. Int J Dev Biol.

[PDF_01113] Tanabe Lorraine, Wilbur W. John (2002). Tagging gene and protein names in biomedical text.. Bioinformatics.

[PDF_00070] Tefferi Ayalew, Bolander Mark E., Ansell Stephen M., Wieben Eric D., Spelsberg Thomas C. (2002). Primer on medical genomics. Part III: Microarray experiments and data analysis.. Mayo Clin Proc.

[PDF_00073] Templin Markus F., Stoll Dieter, Schrenk Monika, Traub Petra C., Vöhringer Christian F., Joos Thomas O. (2002). Protein microarray technology.. Drug Discov Today.

[PDF_00677] Thiede Bernd, Siejak Frank, Dimmler Christiane, Rudel Thomas (2002). Prediction of translocation and cleavage of heterogeneous ribonuclear proteins and Rho guanine nucleotide dissociation inhibitor 2 during apoptosis by subcellular proteome analysis.. Proteomics.

[PDF_00561] Tjaden Brian, Saxena Rini Mukherjee, Stolyar Sergey, Haynor David R., Kolker Eugene, Rosenow Carsten (2002). Transcriptome analysis of Escherichia coli using high-density oligonucleotide probe arrays.. Nucleic Acids Res.

[PDF_01115] Tomida Shuta, Hanai Taizo, Honda Hiroyuki, Kobayashi Takeshi (2002). Analysis of expression profile using fuzzy adaptive resonance theory.. Bioinformatics.

[PDF_00763] Ton Christopher, Stamatiou Dimitri, Dzau Victor J., Liew Choong-Chin (2002). Construction of a zebrafish cDNA microarray: gene expression profiling of the zebrafish during development.. Biochem Biophys Res Commun.

[PDF_00564] Tsangaris George Th, Botsonis Athanassios, Politis Ioannis, Tzortzatou-Stathopoulou Fotini (2002). Evaluation of cadmium-induced transcriptome alterations by three color cDNA labeling microarray analysis on a T-cell line.. Toxicology.

[PDF_01118] Tufféry P. (2002). CS-PSeq-Gen: simulating the evolution of protein sequence under constraints.. Bioinformatics.

[PDF_00766] Unda F. J., Iehara N., De Vega S., De la Fuente M., Vilaxa A., Cobourne M., Sharpe P. T., Yamada Y. (2002). Analysis of cDNAs from a mouse embryo tooth library: identification of novel genes during tooth development.. Connect Tissue Res.

[PDF_00391] Van der Hoeven Rutger, Ronning Catherine, Giovannoni James, Martin Gregory, Tanksley Steven (2002). Deductions about the number, organization, and evolution of genes in the tomato genome based on analysis of a large expressed sequence tag collection and selective genomic sequencing.. Plant Cell.

[PDF_00079] Visca Paolo, Leoni Livia, Wilson Megan J., Lamont Iain L. (2002). Iron transport and regulation, cell signalling and genomics: lessons from Escherichia coli and Pseudomonas.. Mol Microbiol.

[PDF_00855] Vissers Johannes P. C., Blackburn R. Kevin, Moseley M. Arthur (2002). A novel interface for variable flow nanoscale LC/MS/MS for improved proteome coverage.. J Am Soc Mass Spectrom.

[PDF_00681] Wagner Mary Ann, Eschenbrenner Michel, Horn Troy A., Kraycer Jo Ann, Mujer Cesar V., Hagius Sue, Elzer Philip, DelVecchio Vito G. (2002). Global analysis of the Brucella melitensis proteome: Identification of proteins expressed in laboratory-grown culture.. Proteomics.

[PDF_01120] Wang Junbai, Nygaard Vigdis, Smith-Sørensen Birgitte, Hovig Eivind, Myklebost Ola (2002). MArray: analysing single, replicated or reversed microarray experiments.. Bioinformatics.

[PDF_01123] Wang Lichun, Riethoven Jean-Jack, Robinson Alan (2002). XEMBL: distributing EMBL data in XML format.. Bioinformatics.

[PDF_00568] Watanabe Junichi, Sasaki Masahide, Suzuki Yutaka, Sugano Sumio (2002). Analysis of transcriptomes of human malaria parasite Plasmodium falciparum using full-length enriched library: identification of novel genes and diverse transcription start sites of messenger RNAs.. Gene.

[PDF_00354] Watson Mark A., Gutmann David H., Peterson Kelly, Chicoine Michael R., Kleinschmidt-DeMasters Bette K., Brown Henry G., Perry Arie (2002). Molecular characterization of human meningiomas by gene expression profiling using high-density oligonucleotide microarrays.. Am J Pathol.

[PDF_00858] Wattenberg Andreas, Organ Andrew J., Schneider Klaus, Tyldesley Richard, Bordoli Robert, Bateman Robert H. (2002). Sequence dependent fragmentation of peptides generated by MALDI quadrupole time-of-flight (MALDI Q-TOF) mass spectrometry and its implications for protein identification.. J Am Soc Mass Spectrom.

[PDF_00573] Weber Arnim, Jung Kirsten (2002). Profiling early osmostress-dependent gene expression in Escherichia coli using DNA macroarrays.. J Bacteriol.

[PDF_00576] Weindruch Richard, Kayo Tsuyoshi, Lee Cheol Koo, Prolla Tomas A. (2002). Gene expression profiling of aging using DNA microarrays.. Mech Ageing Dev.

[PDF_00579] Weston G. C., Haviv I., Rogers P. A. W. (2002). Microarray analysis of VEGF-responsive genes in myometrial endothelial cells.. Mol Hum Reprod.

[PDF_00358] Wikman Harriet, Kettunen Eeva, Seppänen Jouni K., Karjalainen Antti, Hollmén Jaakko, Anttila Sisko, Knuutila Sakari (2002). Identification of differentially expressed genes in pulmonary adenocarcinoma by using cDNA array.. Oncogene.

[PDF_00685] Willemoës Martin, Kilstrup Mogens, Roepstorff Peter, Hammer Karin (2002). Proteome analysis of a Lactococcus lactis strain overexpressing gapA suggests that the gene product is an auxiliary glyceraldehyde 3-phosphate dehydrogenase.. Proteomics.

[PDF_00426] Winssinger Nicolas, Ficarro Scott, Schultz Peter G., Harris Jennifer L. (2002). Profiling protein function with small molecule microarrays.. Proc Natl Acad Sci U S A.

[PDF_00429] Wu Lani F., Hughes Timothy R., Davierwala Armaity P., Robinson Mark D., Stoughton Roland, Altschuler Steven J. (2002). Large-scale prediction of Saccharomyces cerevisiae gene function using overlapping transcriptional clusters.. Nat Genet.

[PDF_00863] Xiang Charlie C., Kozhich Olga A., Chen Mei, Inman Jason M., Phan Quang N., Chen Yidong, Brownstein Michael J. (2002). Amine-modified random primers to label probes for DNA microarrays.. Nat Biotechnol.

[PDF_00136] Xiao Ming, Zhu Zhi Zhan, Liu Jueping, Zhang Chu Yu (2002). A new method based on entropy theory for genomic sequence analysis.. Acta Biotheor.

[PDF_00582] Xu Qiang, Modrek Barmak, Lee Christopher (2002). Genome-wide detection of tissue-specific alternative splicing in the human transcriptome.. Nucleic Acids Res.

[PDF_01128] Xuan Zhenyu, McCombie W. Richard, Zhang Michael Q. (2002). GFScan: a gene family search tool at genomic DNA level.. Genome Res.

[PDF_00585] Yajima Noriyuki, Masuda Masahisa, Miyazaki Masaru, Nakajima Nobuyuki, Chien Shu, Shyy John Y-J (2002). Oxidative stress is involved in the development of experimental abdominal aortic aneurysm: a study of the transcription profile with complementary DNA microarray.. J Vasc Surg.

[PDF_00082] Yang Yee Hwa, Speed Terry (2002). Design issues for cDNA microarray experiments.. Nat Rev Genet.

[PDF_00366] Yao Ruisheng, Wang Yian, Lubet Ronald A., You Ming (2002). Differentially expressed genes associated with mouse lung tumor progression.. Oncogene.

[PDF_00084] Yarmush Martin L., Jayaraman Arul (2002). Advances in proteomic technologies.. Annu Rev Biomed Eng.

[PDF_01130] Yeramian Edouard, Bonnefoy Serge, Langsley Gordon (2002). Physics-based gene identification: proof of concept for Plasmodium falciparum.. Bioinformatics.

[PDF_00866] Yergey Alfred L., Coorssen Jens R., Backlund Peter S., Blank Paul S., Humphrey Glen A., Zimmerberg Joshua, Campbell Jennifer M., Vestal Marvin L. (2002). De novo sequencing of peptides using MALDI/TOF-TOF.. J Am Soc Mass Spectrom.

[PDF_00589] Yoshida Shigeo, Yashar Beverly M., Hiriyanna Suja, Swaroop Anand (2002). Microarray analysis of gene expression in the aging human retina.. Invest Ophthalmol Vis Sci.

[PDF_01133] Zdobnov Evgeny M., Lopez Rodrigo, Apweiler Rolf, Etzold Thure (2002). The EBI SRS server-new features.. Bioinformatics.

[PDF_00186] Zdobnov Evgeny M., von Mering Christian, Letunic Ivica, Torrents David, Suyama Mikita, Copley Richard R., Christophides George K., Thomasova Dana, Holt Robert A., Subramanian G. Mani (2002). Comparative genome and proteome analysis of Anopheles gambiae and Drosophila melanogaster.. Science.

[PDF_00369] Zhang H. Q., Lu H., Enosawa S., Takahara S., Sakamoto K., Nakajima T., Saito H., Suzuki S. (2002). Microarray analysis of gene expression in peripheral blood mononuclear cells derived from long-surviving renal recipients.. Transplant Proc.

[PDF_00870] Zhang Roujian, Sioma Cathy S., Thompson Robert A., Xiong Li, Regnier Fred E. (2002). Controlling deuterium isotope effects in comparative proteomics.. Anal Chem.

[PDF_00214] Zheng Yu, Szustakowski Joseph D., Fortnow Lance, Roberts Richard J., Kasif Simon (2002). Computational identification of operons in microbial genomes.. Genome Res.

[PDF_00592] Zhou J., Jin Y., Gao Y., Wang H., Hu G., Huang Y., Chen Q., Feng M., Wu C. (2002). Genomic-scale analysis of gene expression profiles in TNF-alpha treated human umbilical vein endothelial cells.. Inflamm Res.

[PDF_01135] Zhou Yan, Huang Guyang Matthew, Wei Liping (2002). UniBLAST: a system to filter, cluster, and display BLAST results and assign unique gene annotation.. Bioinformatics.

[PDF_00689] Zhou Zhaolan, Licklider Lawrence J., Gygi Steven P., Reed Robin (2002). Comprehensive proteomic analysis of the human spliceosome.. Nature.

[PDF_00433] Zwiesler-Vollick Julie, Plovanich-Jones Anne E., Nomura Kinya, Bandyopadhyay Sruti, Joardar Vinita, Kunkel Barbara N., He Sheng Yang (2002). Identification of novel hrp-regulated genes through functional genomic analysis of the Pseudomonas syringae pv. tomato DC3000 genome.. Mol Microbiol.

[PDF_00195] de Haan Gerald, Bystrykh Leonid V., Weersing Ellen, Dontje Bert, Geiger Hartmut, Ivanova Natalia, Lemischka Ihor R., Vellenga Edo, Van Zant Gary (2002). A genetic and genomic analysis identifies a cluster of genes associated with hematopoietic cell turnover.. Blood.

[PDF_00243] de Longueville Françoise, Surry Dominic, Meneses-Lorente Georgina, Bertholet Vincent, Talbot Valérie, Evrard Stephanie, Chandelier Nathalie, Pike Andrew, Worboys Phil, Rasson Jean Paul (2002). Gene expression profiling of drug metabolism and toxicology markers using a low-density DNA microarray.. Biochem Pharmacol.

[PDF_00422] van Dijl Jan Maarten, Braun Peter G., Robinson Colin, Quax Wim J., Antelmann Haike, Hecker Michael, Müller Jörg, Tjalsma Harold, Bron Sierd, Jongbloed Jan D. H. (2002). Functional genomic analysis of the Bacillus subtilis Tat pathway for protein secretion.. J Biotechnol.

[PDF_00770] van Zyl Leonel, von Arnold Sara, Bozhkov Peter, Chen Yongzhong, Egertsdotter Ulrika, Mackay John, Sederoff Ronald R., Shen Jing, Zelena Lyubov, Clapham David H. (2002). Heterologous array analysis in Pinaceae: hybridization of Pinus taeda cDNA arrays with cDNA from needles and embryogenic cultures of P. Taeda, P. Sylvestris or Picea abies.. Comp Funct Genomics.

[PDF_00417] van der Leij Feike R., Cox Keith B., Jackson Vicky N., Huijkman Nicolette C. A., Bartelds Beatrijs, Kuipers Jaap R. G., Dijkhuizen Trijnie, Terpstra Peter, Wood Philip A., Zammit Victor A. (2002). Structural and functional genomics of the CPT1B gene for muscle-type carnitine palmitoyltransferase I in mammals.. J Biol Chem.

